# Structures of a constitutively active mutant of human IDH3 reveal new insights into the mechanisms of allosteric activation and the catalytic reaction

**DOI:** 10.1016/j.jbc.2022.102695

**Published:** 2022-11-12

**Authors:** Xingchen Chen, Pengkai Sun, Yan Liu, Senlin Shen, Tengfei Ma, Jianping Ding

**Affiliations:** 1 State Key Laboratory of Molecular Biology, Shanghai Institute of Biochemistry and Cell Biology, Center for Excellence in Molecular Cell Science, University of Chinese Academy of Sciences, Chinese Academy of Sciences, Shanghai, China; 2 School of Life Science and Technology, ShanghaiTech University, Shanghai, China; 3 School of Life Sciences, Shanghai University, Shanghai, China; 4 School of Life Science, Hangzhou Institute for Advanced Study, University of Chinese Academy of Sciences, Hangzhou, China

**Keywords:** isocitrate dehydrogenase, IDH3, NAD-IDH, protein assembly, allosteric regulation, enzyme catalysis, crystallography, TCA cycle, CIT, citrate, ICT, isocitrate, IDH, isocitrate dehydrogenase, PDB, Protein Data Bank, SEC-MALS, size-exclusion chromatography–multiangle light scattering, TSA, thermal shift assay

## Abstract

Human NAD-dependent isocitrate dehydrogenase or IDH3 (HsIDH3) catalyzes the decarboxylation of isocitrate into α-ketoglutarate in the tricarboxylic acid cycle. It consists of three types of subunits (α, β, and γ) and exists and functions as the (αβαγ)_2_ heterooctamer. HsIDH3 is regulated allosterically and/or competitively by numerous metabolites including CIT, ADP, ATP, and NADH. Our previous studies have revealed the molecular basis for the activity and regulation of the αβ and αγ heterodimers. However, the molecular mechanism for the allosteric activation of the HsIDH3 holoenzyme remains elusive. In this work, we report the crystal structures of the αβ and αγ heterodimers and the (αβαγ)_2_ heterooctamer containing an α-Q139A mutation in the clasp domain, which renders all the heterodimers and the heterooctamer constitutively active in the absence of activators. Our structural analysis shows that the α-Q139A mutation alters the hydrogen-bonding network at the heterodimer-heterodimer interface in a manner similar to that in the activator-bound αγ heterodimer. This alteration not only stabilizes the active sites of both α_Q139A_β and α_Q139A_γ heterodimers in active conformations but also induces conformational changes of the pseudo-allosteric site of the α_Q139A_β heterodimer enabling it to bind activators. In addition, the α_Q139A_^ICT+Ca+NAD^β^NAD^ structure presents the first pseudo-Michaelis complex of HsIDH3, which allows us to identify the key residues involved in the binding of cofactor, substrate, and metal ion. Our structural and biochemical data together reveal new insights into the molecular mechanisms for allosteric regulation and the catalytic reaction of HsIDH3.

Eukaryotic NAD-dependent isocitrate (ICT) dehydrogenases (NAD-IDHs), which are localized to the mitochondria, are responsible for catalyzing the oxidative decarboxylation of ICT into α-ketoglutarate (α-KG) using NAD as coenzyme. This catalytic reaction is the rate-limiting step of the tricarboxylic acid cycle (also called citric acid or Krebs cycle), which is an essential metabolic pathway for carbohydrates, fats, and proteins. Thus, NAD-IDHs play an important role in cell growth and development. In most eukaryotes, NAD-IDHs exist and function as hetero-oligomers, which share a similar catalytic mechanism but distinct regulatory mechanisms in different species. In lower eukaryotes such as *Saccharomyces cerevisiae*, NAD-IDH is composed of a regulatory subunit ScIDH1 and a catalytic subunit ScIDH2 in a 1:1 ratio, which share a sequence identity of 41.6% (1, 2, 3). The two subunits form the ScIDH1/ScIDH2 heterodimer, which assembles into a heterotetramer and further into a heterooctamer (4). Citrate (CIT) and AMP act as activators that bind to the allosteric site in ScIDH1, which causes conformational changes of ScIDH2, leading to activation of the enzyme (4). Mammalian NAD-IDHs (also called IDH3) are a heterooctameric complex composed of three types of subunits in a ratio of 2α:1β:1γ (5, 6, 7). For human IDH3 (HsIDH3), the pairwise sequence identities between α and β, α and γ, and β and γ are 44.6%, 43.6%, and 51.9%, respectively (Fig. S1*A*). The function of IDH3 also requires proper assembly of component subunits. The α, β, and γ subunits form the αβ and αγ heterodimers, which assemble into the αβαγ heterotetramer and further into the (αβαγ)_2_ heterooctamer (8). The (αβαγ)_2_ heterooctamer is also called the holoenzyme. Early biochemical studies of mammalian IDH3s showed that the α subunit is catalytic subunit while the β and γ subunits are regulatory subunits (9, 10, 11, 12, 13). The enzymatic activity of IDH3 can be positively regulated by CIT (14) and ADP (15) and negatively regulated by ATP (16) and NADH (17). The enzymatic activity of IDH3 requires the presence of a divalent metal ion with Mn^2+^ and Mg^2+^ as the most effective ones but Ca^2+^ can inhibit the catalytic reaction (18).

Previously, we carried out biochemical and structural studies of the composing αβ and αγ heterodimers and the (αβαγ)_2_ holoenzyme of HsIDH3 to investigate the functional roles of each subunit and the molecular mechanism for allosteric activation of the enzymes (18, 19, 20, 21, 22). We found that in both αβ and αγ heterodimers, the α subunit possesses catalytic activity; but, only the γ subunit plays a regulatory role in an allosteric manner, while the β subunit plays a structural role to facilitate the assembly of the holoenzyme (18). In the absence of activators, both αβ and αγ heterodimers exhibit weak catalytic activity (basal activity). The αγ heterodimer can be activated by CIT and ADP, whereas the αβ heterodimer cannot be activated (18). The crystal structures of the αγ and αβ heterodimers revealed the underlying molecular mechanisms (19, 20, 21, 22). Specifically, the γ subunit contains an allosteric site that can bind CIT and ADP (19). The binding of CIT and ADP induces conformational changes of the γ subunit, which remodel hydrogen-bonding network at the α-γ interface and induce conformational changes of the active site to adopt an active conformation favorable for substrate (ICT) binding, leading to the activation of the αγ heterodimer (19). In contrast, the β subunit contains a pseudo-allosteric site, which is structurally unable to bind CIT or ADP, and thus the αβ heterodimer cannot be allosterically activated (21).

Recently, we also solved the crystal structure of the HsIDH3 holoenzyme in unliganded form, which revealed the assembly of the (αβαγ)_2_ heterooctamer (Fig. S1*B*) (23). Specifically, the α, β, and γ subunits all consist of a large domain, a small domain, and a clasp domain. The α and β subunits form the αβ heterodimer and the α and γ subunits form the αγ heterodimer. The αβ and αγ heterodimers assemble into the αβαγ heterotetramer *via* their clasp domains, and two heterotetramers further assemble into the (αβαγ)_2_ heterooctamer with the N terminus of the γ subunit of one heterotetramer inserted into the β subunit of the other heterotetramer (Fig. S1*B*) (23). In the (αβαγ)_2_ heterooctamer, the two heterotetramers are arranged in perpendicular to each other with a tetrahedron architecture; the two β and two γ subunits are arranged alternately to form the inner core, and the four α subunits are positioned on the periphery (Fig. S1*B*). In this structure, both αγ and αβ heterodimers adopt inactive conformations and thus the holoenzyme assumes an inactive conformation. Interestingly, in both the absence and presence of the activators, the (αβαγ)_2_ holoenzyme exhibits much higher activities and tighter binding affinities for ICT than those of the isolated αβ and αγ heterodimers, suggesting that there is a synergistic effect between the αβ and αγ heterodimers in the holoenzyme (23). Therefore, it was postulated that in the holoenzyme, both α subunits could be activated and the γ subunit might regulate the α subunits in both αβ and αγ heterodimers through an allosteric mechanism (18, 23). However, the molecular basis for the allosteric activation of the holoenzyme, especially for how the activation signal would be transmitted from the γ subunit to the α subunit in the αβ heterodimer, is still unclear. To this end, we performed crystallographic studies of the HsIDH3 holoenzyme in the presence of various activators and ligands. Although we were able to grow crystals of decent sizes, unfortunately these crystals diffracted X-rays only to low resolutions (about 6–7 Å), prohibiting us from obtaining a high-resolution crystal structure of the activator-bound HsIDH3 holoenzyme with an active conformation.

Meanwhile, during the examination of the functional roles of numerous key residues in the activation of the HsIDH3 holoenzyme, we found that intriguingly, the mutant holoenzymes containing the Q139A mutation in the clasp domain of the α subunit (α_Q139A_ or α_M_), namely the (α_M_βαγ)_2_, (αβα_M_γ)_2_, and (α_M_βα_M_γ)_2_ holoenzymes, are all constitutively active (23). α-Gln139 is involved in the intersubunit hydrogen-bonding interactions in the structures of the isolated αβ and αγ heterodimers and the unliganded (αβαγ)_2_ holoenzyme (19, 21, 23). The hydrogen-bonding network at the α-γ interface and the αγ-αγ interface is remodeled during the activation of the αγ heterodimer (19). These results prompted us to speculate that α-Gln139 might play an important role in the allosteric activation of the HsIDH3 holoenzyme. In this work, we carried out biochemical and structural studies of the α_M_β, α_M_γ, and α_M_βα_M_γ mutant enzymes. We found that like the α_M_-containing holoenzymes, the α_M_β and α_M_γ heterodimers are also constitutively active. To understand the underlying molecular mechanism, we determined the crystal structures of the α_M_β and α_M_γ heterodimers in both unliganded and ligand-bound forms and a moderate-resolution crystal structure of the (α_M_βα_M_γ)_2_ holoenzyme in unliganded form. Structural analysis shows that consistent with the biochemical data, the structures of both α_M_β and α_M_γ heterodimers assume active conformations regardless the absence or presence of activators. In addition, the structure of the (α_M_βα_M_γ)_2_ holoenzyme also assumes an active conformation as indicated by the conformations of several key structure elements, despite the absence of activators. The α-Q139A mutation alters the hydrogen-bonding network at the heterodimer-heterodimer interface in a similar manner as that in the activation of the αγ heterodimer, which not only stabilizes the active sites of both α_M_β and α_M_γ heterodimers in active conformations but also induces conformational changes of the pseudo-allosteric site of the α_M_β heterodimer enabling it to bind activators. Moreover, the crystal structure of α_M_^ICT+Ca+NAD^β^NAD^ presents the first pseudo-Michaelis complex for HsIDH3 and other NAD-IDHs, which allows us to identify the key residues involved in the binding of cofactor, substrate, and metal ion. Our structural and biochemical data together reveal new insights into the molecular mechanisms for the allosteric activation and the catalytic reaction of HsIDH3. These findings provide the guidance for further study of the structure and function relationship and allosteric regulation mechanism of other mammalian IDH3 and allosterically regulated enzymes, as well as the molecular basis and technical tool for further investigation of the functional roles of disease-associated mutations and/or dysregulation of the component subunits of HsIDH3 in the pathogenesis of relevant diseases.

## Results

### The α_M_β and α_M_γ heterodimers are constitutively active

Our previous biochemical data showed that the WT αβ and αγ heterodimers and (αβαγ)_2_ holoenzyme exhibit low *V*_max_ (the maximal velocity of the enzymatic reaction) and high *S*_0.5_ for ICT (*S*_0.5,ICT_; the substrate concentration at half-maximum velocity, which approximates the apparent Michaelis constant *K*_m_) in the absence of activators (basal activity) (Table 1 and Fig. S2) (18, 21). In the presence of CIT and ADP, the activities of the WT αγ and (αβαγ)_2_ are potentiated by 20.8-fold and 8.2-fold (defined as the ratio of *S*_0.5,ICT_ (−activators)/*S*_0.5,ICT_ (+activators)), respectively, which are manifested by moderately increased *V*_max_ and significantly decreased *S*_0.5,ICT_. In contrast, the WT αβ heterodimer shows unchanged *V*_max_ and *S*_0.5,ICT_ in the absence and presence of activators, indicating that it could not be activated. Intriguingly, all of the (α_M_βαγ)_2_, (αβα_M_γ)_2_, and (α_M_βα_M_γ)_2_ mutant holoenzymes are constitutively active in the absence of activators. To investigate the functional role of the α-Q139A mutation in the activation of these mutant holoenzymes, we expressed and purified the α_M_β and α_M_γ heterodimers and measured their kinetic parameters in the absence and presence of activators (CIT and ADP). The results show that in the absence of activators, both α_M_β and α_M_γ heterodimers exhibit significantly higher *V*_max_ and lower *S*_0.5,ICT_ compared to those of the WT αβ and αγ heterodimers (Table 1 and Fig. S2). Specifically, α_M_β shows a *V*_max_ of 18.0 ± 1.4 μmol∙min^−1^ mg^−1^ and a *S*_0.5,ICT_ of 0.17 ± 0.02 mM, and α_M_γ shows a *V*_max_ of 25.5 ± 2.3 μmol∙min^−1^ mg^−1^ and a *S*_0.5,ICT_ of 0.26 ± 0.11 mM. Notably, the binding affinities of α_M_β and α_M_γ for ICT (reflected by *S*_0.5,ICT_) in the absence of activators are comparable to those of WT αγ in the presence of activators (*p* values > 0.09) (Fig. S2*D*). Addition of the activators confers only slight activation effect (1.7–2.0-fold) for the mutant heterodimers. These results indicate that like the mutant holoenzymes, the α_M_β and α_M_γ heterodimers are constitutively active regardless the absence or presence of activators.

**Table 1:** Kinetic parameters of the WT and mutant HsIDH3^a^

Enzyme	−Activators		+Activators (CIT and ADP)		Activation effect (fold)	Reference
	*V*_max_ (μmol∙min^−1^ mg^−1^)	*S*_0.5,ICT_ (mM)	*V*_max_ (μmol∙min^−1^ mg^−1^)	*S*_0.5,ICT_ (mM)		
αβ	2.72 ± 0.14	3.65 ± 0.39	2.80 ± 0.23	3.63 ± 0.62	1.0	(23)
αγ	9.62 ± 0.23	5.42 ± 0.71	16.1 ± 1.1	0.26 ± 0.07	20.8	(23)
(αβαγ)_2_	28.6 ± 0.3	3.54 ± 0.18	30.6 ± 1.0	0.43 ± 0.03	8.2	(23)
α_M_β	18.0 ± 1.4	0.17 ± 0.02	19.3 ± 0.5	0.10 ± 0.06	1.7	This work
α_M_γ	25.5 ± 2.3	0.26 ± 0.11	26.6 ± 2.3	0.13 ± 0.08	2.0	This work
(α_M_βα_M_γ)_2_	39.7 ± 0.9	0.24 ± 0.03	47.4 ± 1.6	0.18 ± 0.03	1.3	(23)

a Kinetic parameters of the WT and α-Q139A (α_M_) containing mutants of αβ, αγ, and (αβαγ)_2_ in the absence and presence of activators (ADP and CIT) are determined. *V*_max_ is the maximal velocity of the enzymatic reaction when the enzyme is saturated by the substrate. *S*_0.5,ICT_ is the substrate concentration at half-maximal velocity, which approximates the apparent Michaelis constant *K*_m_. Results are presented as the mean ± SD from three independent experiments. Activation effect (fold) = *S*_0.5,ICT_ (−activators)/*S*_0.5,ICT_ (+activators).

**Figure 1:**
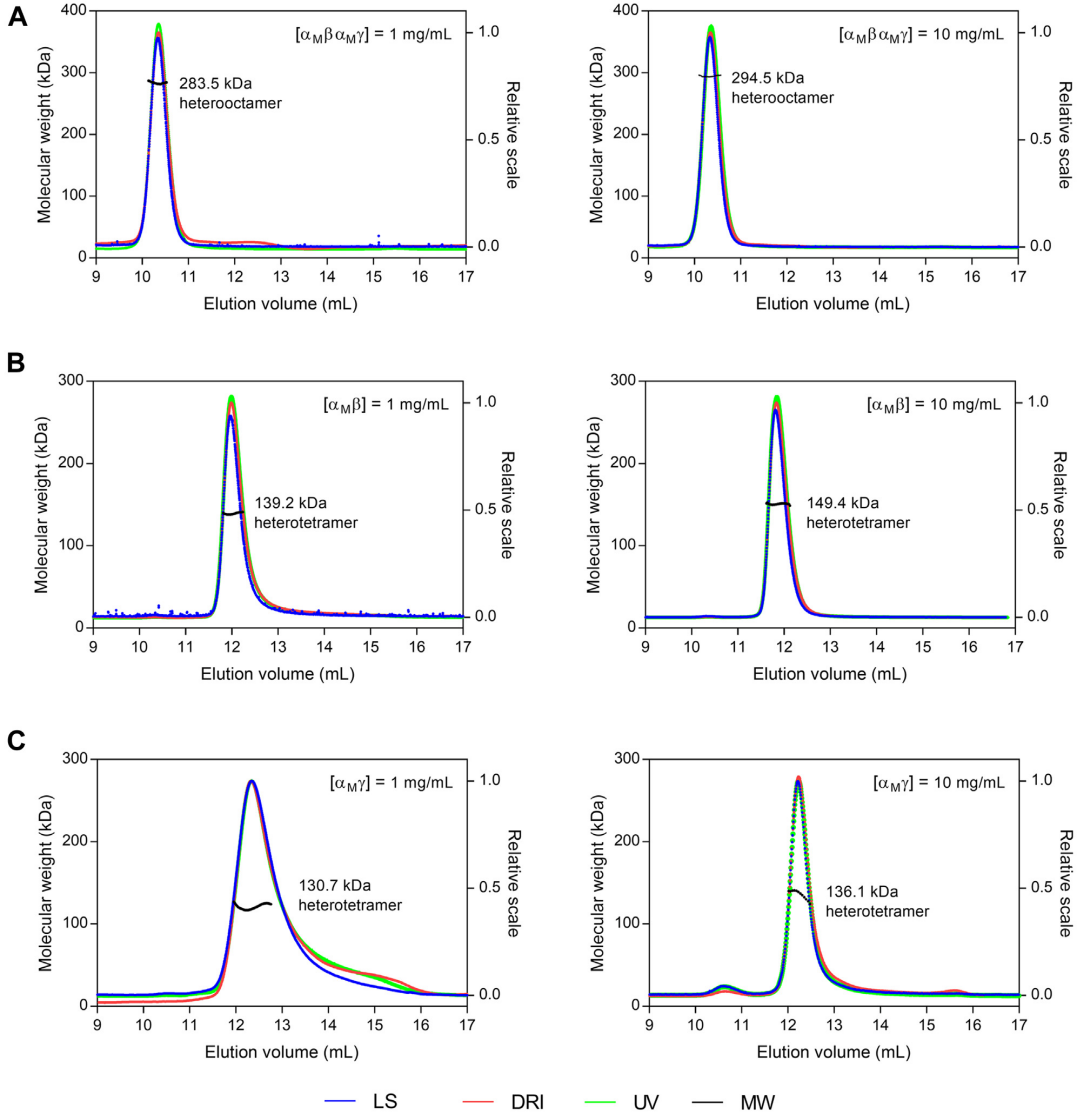
**SEC-MALS analysis of the α-Q139A mutation containing HsIDH3.** (*A*) α_M_βα_M_γ, (*B*) α_M_β, and (*C*) α_M_γ. These mutant enzymes were measured at a low concentration (1 mg/ml, left panel) and a high concentration (10 mg/ml, right panel). Chromatograms show the molecular weight (MW, *black*), and the normalized *curves* of the light scattering at 90° (LS, *blue*), differential refractive index (DRI, *red*) and UV (*green*). All experiments were performed twice, which yielded similar results, and for each case, only the data of one representative experiment is shown. SEC-MALS, size-exclusion chromatography–multiangle light scattering.

Moreover, the size-exclusion chromatography–multiangle light scattering (SEC-MALS) analysis shows that similar to the WT holoenzyme, the mutant holoenzyme displays an average mass of approximately 290 kDa at both low concentration (1 mg/ml) and high concentration (10 mg/ml) corresponding to an (α_M_βα_M_γ)_2_ heterooctamer (Fig. 1). In contrast to the WT αβ and αγ heterodimers, both of which exist as heterodimers (75 kDa) at low concentration and a dimer of heterodimers or heterotetramer [(αβ)_2_ and (αγ)_2_] (130 kDa) at high concentration (18), the mutant heterodimers exhibit an average molecular mass of approximately 131 to 149 kDa at both low and high concentrations, corresponding to a heterotetramer [(α_M_β)_2_ and (α_M_γ)_2_] (Fig. 1). In addition, the thermal shift assay (TSA) analysis shows that the melting temperatures (*T*_m_) of α_M_β and α_M_γ at low concentration (1 mg/ml) are ∼5 °C higher than that of WT αβ and αγ, indicating that α_M_β and α_M_γ are more stable than WT αβ and αγ (Fig. S3). These results indicate that the α-Q139A mutation stabilizes the heterotetrameric forms of α_M_β and α_M_γ.

### Crystal structures of the α_M_γ and α_M_β heterodimers

To understand the molecular basis for the functional role of the α-Q139A mutation in the constitutive activation and stabilization of the heterotetrameric forms of α_M_γ and α_M_β, we determined the crystal structures of α_M_γ in apo form (α_M_γ) and a ligand-bound form (α_M_γ^CIT^) and α_M_β in apo form (α_M_β) and two ligand-bound forms (α_M_^ICT+Ca+NAD^β^NAD^ and α_M_^Mg^β^ADP^) (Table 2). Crystals of the apo α_M_γ belong to space group *C*2, and the asymmetry unit contains one α_M_γ heterodimer, which forms an (α_M_γ)_2_ heterotetramer with a crystallographic 2-fold axis-related heterodimer (Table 2). Crystals of α_M_γ^CIT^ belongs to space group *P*3_1_21, and the asymmetry unit contains one α_M_γ heterodimer, which also forms an (α_M_γ)_2_ heterotetramer with a crystallographic 2-fold axis-related heterodimer (Table 2). In this structure, there is a CIT bound at the allosteric site in the γ subunit with well-defined electron density.

**Table 2:** Statistics of diffraction data and structure refinement

Structure	α_M_β	α_M_^ICT+Ca+NAD^β^NAD^	α_M_^Mg^β^ADP^	α_M_γ	α_M_γ^CIT^	(α_M_βα_M_γ)_2_
Diffraction data						
Wavelength (Å)	0.9786	0.9786	0.9785	0.9792	0.9778	0.9793
Space group	*C*2	*C*222_1_	*I*422	*C*2	*P*3_1_21	*C*222_1_
Cell parameters						
a (Å)	209.15	134.04	165.90	137.51	111.99	159.26
b (Å)	170.03	163.83	165.90	73.53	111.99	490.33
c (Å)	208.69	163.13	129.90	86.62	145.93	330.46
α (°)	90	90	90	90	90	90
β (°)	103.11	90	90	107.36	90	90
γ (°)	90	90	90	90	120	90
Resolution (Å)	50.0–2.85 (2.95–2.85)^a^	50.0–2.85 (2.95–2.85)	50.0–2.70 (2.75–2.70)	50.0–2.70 (2.80–2.70)	50.0–2.50 (2.59–2.50)	50.0–4.50 (4.66–4.50)
Observed reflections	1,140,342	540,299	660,435	166,439	703,070	279,482
Unique reflections (I/σ (I)>0)	165,707	42,112	25,101	22,588	36,769	69,186
Average redundancy	6.9 (6.8)	12.8 (11.2)	26.3 (26.0)	7.4 (7.5)	19.1 (13.4)	4.0 (4.0)
Average I/σ (I)	11.3 (2.0)	11.4 (2.1)	17.7 (2.5)	22.6 (6.9)	24.3 (1.9)	5.7 (1.3)
Completeness (%)	100.0 (100.0)	99.9 (99.9)	100.0 (99.9)	99.8 (99.9)	99.7 (97.4)	89.3 (92.1)
R_merge_ (%)^b^	15.0 (73.7)	17.9 (100.2)	14.9 (108.6)	12.3 (48.8)	12.3 (75.7)	20.3 (80.9)
CC_½_ (%)	99.6 (83.5)	100.0 (83.6)	101.3 (90.4)	97.7 (94.7)	95.3 (96.7)	96.8 (60.6)
Refinement and structure model						
No. of reflections (*Fo*>0σ (*Fo*))	165,383	42,004	24,914	22,577	36,395	65,344
Working set	157,013	39,838	23,666	21,442	34,551	62,098
Test set	8370	2166	1248	1135	1844	3246
R_work_/R_free_ factor^c^	0.20/0.25	0.21/0.26	0.19/0.24	0.19/0.24	0.22/0.26	0.27/0.29
No. of nonhydrogen atoms	39,057	10,093	5094	4929	5085	39,376
No. of molecules/ASU	8	2	1	1	1	2
Average B factor (Å^2^)	52.7	44.5	50.2	49.7	79.1	107.5
Protein	52.7	44.3	50.2	49.7	79.1	107.5
Ligand	-	53.2	44.0	-	67.6	-
Metal ion	-	70.5	32.8	-	-	-
Water	-	-	48.0	46.3	74.8	-
RMSD						
Bond lengths (Å)	0.014	0.010	0.009	0.011	0.008	0.007
Bond angles (°)	1.1	1.3	1.0	1.2	1.0	1.2
Ramachandran plot (%)						
Most favored	93.8	95.3	95.3	96.8	93.2	88.0
Allowed	6.2	4.7	4.7	3.2	6.8	12.4
Disallowed	0	0	0	0	0	0.6

a Numbers in parentheses refer to the highest resolution shell.

b $R_{\text{merge}}=\sum_{hkl}\sum_{i}\left|I_{j}\;{\left(hkl\right)}_{i}-<I\left(\left(hkl\right)\right)>/\sum_{hkl}\sum_{i}I_{i}\left(hkl\right)\right|$.

c $R\;\text{factor}=\sum \left|\left|F_{o}\right|-\left|F_{c}\right|\right|/\sum \left|F_{o}\right|$.

Crystals of the apo α_M_β belong to space group *C*2_1_, and the asymmetry unit contains eight α_M_β heterodimers, which form four (α_M_β)_2_ heterotetramers through noncrystallographic 2-fold axes (Table 2). Crystals of α_M_^Mg^β^ADP^ belong to space group *I*422, and the asymmetry unit contains one α_M_β heterodimer, which forms an (α_M_β)_2_ heterotetramer with a crystallographic 2-fold axis-related heterodimer (Table 2). In this structure, there is an Mg^2+^ ion bound at the active site in the α subunit and an ADP bound at the pseudo-allosteric site in the β subunit, which are well defined in the electron density map. Crystals of α_M_^ICT+Ca+NAD^β^NAD^ belong to space group *C*222_1_ and there are two α_M_β heterodimers in an asymmetry unit, which form an (α_M_β)_2_ heterotetramer through a noncrystallographic 2-fold axis (Table 2). In this structure, there are an ICT, NAD, and Ca^2+^ bound at the active site of the α subunit, which are clearly defined in the electron density map; in addition, there is an NAD bound at the pseudo-allosteric site of the β subunit, and the ADP-ribose moiety of NAD is well defined in the electron density map albeit the nicotinamide moiety is poor defined. In the aforementioned two structures, the bound metal ion at the active site was interpreted as Mg^2+^ or Ca^2+^ due to the presence of Mg^2+^ or Ca^2+^ in the crystallization solution, respectively. These structures were refined to a resolution in the range of 2.50 to 2.85 Å, and most of the polypeptide chains are well defined except for a few surface loops and several residues at the N and C termini. Statistics of the diffraction data, structure refinement, and structure models of these structures are summarized in Table 2.

**Figure 2:**
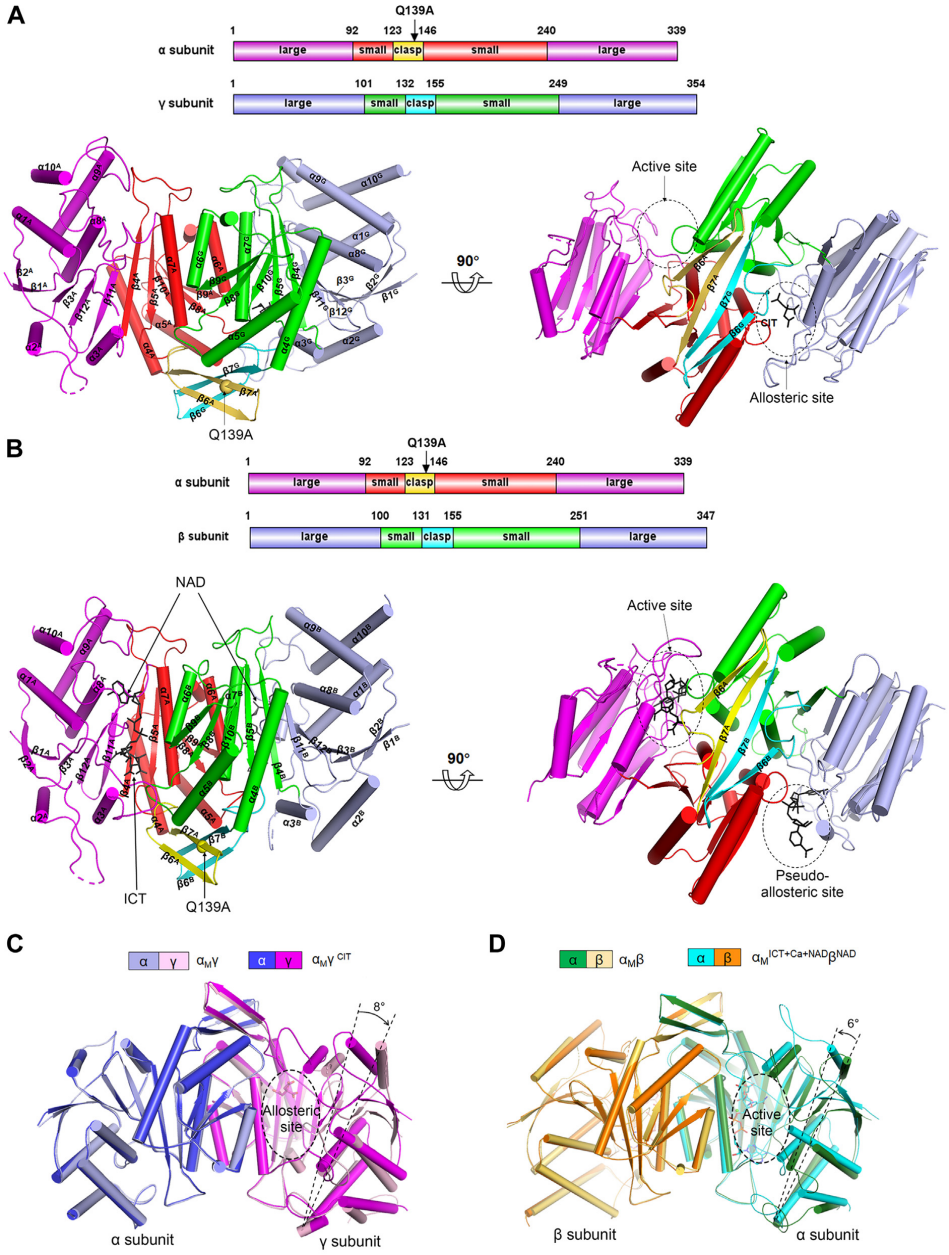
**Structures of the α**_**M**_**γ and α**_**M**_**β heterodimers.***A*, overall structure of α_M_γ^CIT^. *B*, overall structure of α_M_^ICT+Ca+NAD^β^NAD^. Structural elements of the α, β, and γ subunits are superscripted as “A,” “B,” and “G”, respectively. *C*, structural comparison of α_M_γ and α_M_γ^CIT^. Superposition of α_M_γ and α_M_γ^CIT^ reveals a major conformational difference of the γ subunit. *D*, structural comparison of α_M_β and α_M_^ICT+Ca+NAD^β^NAD^. The active site adopts an “open” conformation in α_M_β but a “closed” conformation in α_M_^ICT+Ca+NAD^β^NAD^ due to the movement of the α large domain toward the α-β interface (indicated by an *arrow*). The orientations of the αγ heterodimer in (*C*) and the αβ heterodimer in (*D*) are related by 180° rotation in order to show the allosteric site and the active site at the front, respectively.

Like in the WT αγ and αβ structures, in the α_M_γ and α_M_β structures, each of the α, β, and γ subunits consist of a large domain, a small domain, and a clasp domain (Fig. 2, *A* and *B*). The active site is located in a cleft formed by the α large domain and the β/γ small domain; the allosteric site is formed by the γ large domain and the α small domain; and the pseudo-allosteric site is formed by the β large domain and the α small domain. The α-Q139A mutation is located in the β7 strand of the α clasp domain.

Structural comparison shows that the overall structure of α_M_γ^CIT^ is very similar to that of α^Mg^γ (Protein Data Bank [PDB] 5GRH) and α^Mg^γ^Mg+CIT^ (PDB 5GRI) (RMSD of 0.3 Å and 1.1 Å for about 640 Cα atoms, respectively); however, the overall structure of α_M_γ shows some conformational differences from that of α_M_γ^CIT^ (Fig. 2*C*). In the α_M_γ^CIT^, α^Mg^γ, and α^Mg^γ^Mg+CIT^ structures, the allosteric site always assumes a “closed” conformation. However, in the α_M_γ structure, the γ large domain exhibits an approximate 8° outward rotation from the α-γ interface compared to that in the α_M_γ^CIT^ structure, resulting in an “open” conformation of the allosteric site (Fig. 2*C*). Crystal packing analyses show that in the α_M_γ structure, the γ large domain is involved in close contacts (<4 Å) with the α large domain of a symmetry-related heterodimer and thus its conformation appears to be constrained by the crystal packing (Fig. S4); whereas, in the α_M_γ^CIT^, α^Mg^γ, and α^Mg^γ^Mg+CIT^ structures (which all adopt the same space group), the γ large domain is not involved in crystal packing and thus its conformation is not constrained. Nevertheless, we cannot exclude the possibility that the conformational flexibility of the γ large domain may play a role in the proper function and catalytic reaction of the αγ heterodimer and the holoenzyme.

On the other hand, the overall structures of α_M_β and α_M_^Mg^β^ADP^ are similar to that of the apo αβ (PDB 6KDF), indicated by an RMSD of 0.25 Å and 1.1 Å for about 650 Cα atoms, respectively. However, the overall structure of α_M_^ICT+Ca+NAD^β^NAD^ shows substantial conformational differences from those of αβ, α_M_β, and α_M_^Mg^β^ADP^ (RMSD of 1.5–2.4 Å for about 650 Cα atoms). In the αβ, α_M_β, and α_M_^Mg^β^ADP^ structures, the active site assumes an “open” conformation due to the absence of substrate and/or cofactor. In contrast, compared to that in the α_M_β structure, the α large domain in the α_M_^ICT+Ca+NAD^β^NAD^ structure exhibits a 6° rotation toward the α-β interface owing to the binding of ICT and NAD, resulting in a “closed” conformation of the active site (see Discussion later) (Fig. 2*D*).

### The WT αγ and αβ heterodimers form heterotetramers in the crystal structures to mimic the assembly of the αβαγ heterotetramer

**Figure 3:**
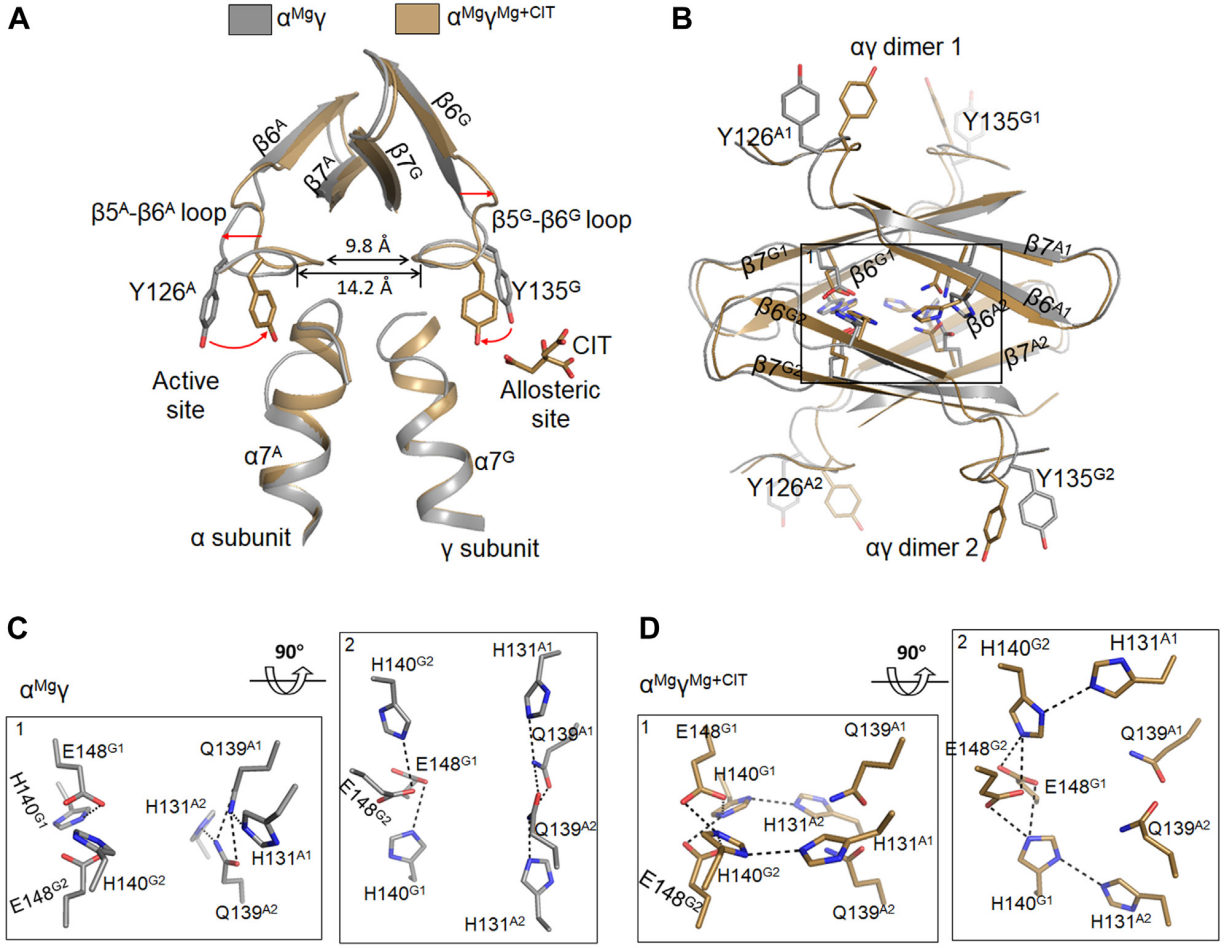
**The WT αγ heterodimer shows distinct structural features in the active and inactive states.***A*, comparison of α^Mg^γ (PDB 5GRH) in the inactive state and α^Mg^γ^Mg+CIT^ (PDB 5GRI) in the active state shows that the α7 helices and β5-β6 loops assume different conformations. Structural elements of the α and γ subunits are superscripted as “A” and “G,” respectively. *Red arrows* indicate the conformational changes of the key residues/structure elements upon activation. *B*, comparison of the αγ-αγ interface in the α^Mg^γ and α^Mg^γ^Mg+CIT^ structures. Heterodimer 1 and heterodimer 2 are related by crystallographic 2-fold axis and form a heterotetramer *via* the clasp domains. Structure elements of the αγ heterodimers 1 and 2 are superscripted as “A1” and “G1” and “A2” and “G2”, respectively. Key residues involved in the rearrangement of hydrogen-bonding network are shown in stick model. *C*, zoom-in views of the hydrogen-bonding network at the αγ-αγ interface in the inactive α^Mg^γ structure. *D*, zoom-in views of the hydrogen-bonding network at the αγ-αγ interface in the active α^Mg^γ^Mg+CIT^ structure. Hydrogen bonds are indicated by *black dash lines*. PDB, Protein Data Bank.

Previously, we determined the crystal structures of the WT αβ and αγ heterodimers in apo form and various ligand-bound forms, which adopt essentially two types of overall conformations: either inactive or active conformation (19, 21, 22). Comparison of the typical inactive α^Mg^γ and active α^Mg^γ^Mg+CIT^ structures reveals substantial conformational changes in both subunits and at the α-γ heterodimer interface (19). Specifically, upon the CIT binding to the allosteric site, the sidechain of Tyr135^G^ (residues and structural elements of the α, β, and γ subunits are superscripted as “A,” “B,” and “G,” respectively) deflects toward the allosteric site and forms a hydrogen bond with the γ-carboxyl group of CIT; the Tyr135^G^-containing β5^G^-β6^G^ loop moves toward the α-γ interface; and the N-terminal region of the α7^G^ helix transforms from a loop conformation into a helical conformation (Fig. 3*A*). These conformational changes are accompanied with the alteration of hydrogen-bonding interactions at the allosteric site and the α-γ interface. Consequently, the β5^A^-β6^A^ loop and the N-terminal region of the α7^A^ helix of the α subunit undergo similar conformational changes in a pseudo-symmetric manner, and the sidechain of Tyr126^A^ in the β5^A^-β6^A^ loop rotates toward the active site and adopts a conformation favorable for substrate binding (Fig. 3*A*). In the αβ structures, the pseudo-allosteric site is occupied by the β3^B^-α3^B^ and β12^B^-α8^B^ loops and thus is unable to bind the activators, explaining why the αβ heterodimer cannot be allosterically regulated by the activators (Fig. S5) (18, 21).

It is interesting to observe that although the WT αβ and αγ heterodimers exist as dimers at low concentrations and tetramers at high concentrations in solution, they always form dimers of heterodimers or heterotetramers [(αβ)_2_ and (αγ)_2_] with crystallographic 2-fold axis-related heterodimers in the crystal structures. In the (αβ)_2_ and (αγ)_2_ heterotetramers, the heterodimer-heterodimer (αβ-αβ and αγ-αγ) interfaces are mediated by their clasp domains (Fig. 3*B*) (23). Detailed structural analyses of the inactive α^Mg^γ and active α^Mg^γ^Mg+CIT^ heterotetramers reveal that the CIT-binding induced conformational changes and in particular the movements of the β5-β6 loops also cause conformational changes of the β6 and β7 strands of the clasp domains, leading to rearrangement of the hydrogen-bonding network at the αγ-αγ interface (Fig. 3, *B*–*D*). Specifically, in the inactive state, the sidechain of Gln139^A1^ forms hydrogen bonds with the sidechains of Gln139^A2^ and His131^A1^, and the sidechain of His140^G1^ forms a hydrogen bond with the sidechain of Glu148^G1^ and vice versa for those of Gln139^A2^ and His140^G2^ (Fig. 3*C*; superscripts “1” and “2” are used to distinguish residues of the two αγ heterodimers forming the heterotetramer). Upon the CIT binding, the Gln139^A1^-Gln139^A2^, His131^A1^-Gln139^A1^, and His131^A2^-Gln139^A2^ hydrogen bonds are disrupted; instead, the sidechain of His131^A1^ forms a new hydrogen bond with the sidechain of His140^G2^, and the sidechain of His140^G1^ forms an additional hydrogen bond with the sidechain of Glu148^G2^ and vice versa for His131^A2^ and His140^G2^ (Fig. 3*D*).

Further structural comparison shows that the formations of the (αβ)_2_ and (αγ)_2_ heterotetramers *via* their clasp domains are in similar manner as the assembly of the αβ and αγ heterodimers into the αβαγ heterotetramer in the unliganded HsIDH3 holoenzyme (Fig. S6*A*) (23). In particular, the hydrogen-bonding networks at the αβ-αβ interface in the inactive α^NAD^β structure (Fig. S6*B*) and at the αβ-αγ interface in the inactive (αβαγ)_2_ structure (Fig. S6*C*) are formed in the same manner as that at the αγ-αγ interface in the inactive α^Mg^γ structure (Fig. 3*C*). Specifically, in the inactive α^NAD^β structure, the sidechain of Gln139^A1^ forms hydrogen bonds with the sidechains of Gln139^A2^ and His131^A1^ and the sidechain of His142^B1^ (corresponding to His140^G^) forms a hydrogen bond with the sidechain of Glu150^B2^ (corresponding to Glu148^G^) and vice versa for those of Gln139^A2^ and His142^B2^ (Fig. S6*B*). In the inactive (αβαγ)_2_ structure, the sidechain of Gln139^A1^ forms hydrogen bonds with the sidechains of Gln139^A2^ and His131^A1^ and vice versa for those of Gln139^A2^; besides, the sidechain of His142^B^ forms a hydrogen bond with the sidechain of Glu150^B^ and sidechain of His140^G^ forms a hydrogen bond with the sidechain of Glu148^G^ (Fig. S6*C*).

These results suggest that the (αβ)_2_ and (αγ)_2_ heterotetramers might mimic the αβαγ heterotetramer and the rearrangement of hydrogen-bonding network at the heterodimer-heterodimer interface in the activation of the αβ and αγ heterodimers might mimic that at the αβ-αγ interface in the activation of the αβαγ heterotetramer. These results further prompt us to speculate that in the αβαγ heterotetramer, there is structural communication between the αβ and αγ heterodimers through the clasp domains, and the CIT-binding induced rearrangement of hydrogen-bonding network at the heterodimer-heterodimer interface plays an important role in the allosteric regulation of the αβαγ heterotetramer and the holoenzyme.

### Structures of the α_M_β and α_M_γ heterodimers all assume active conformations

**Figure 4:**
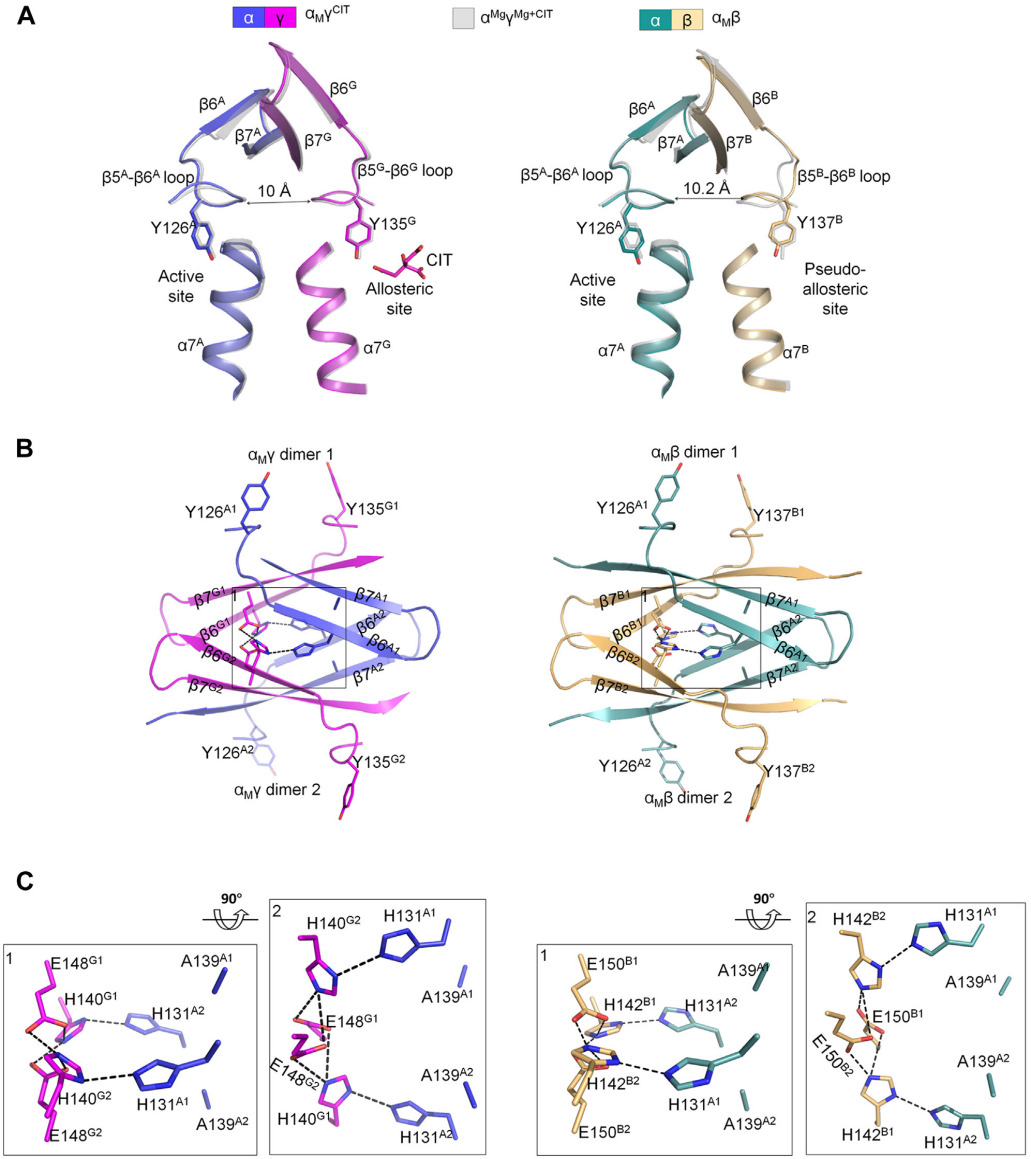
**The overall structures of α**_**M**_**γ and α**_**M**_**β assume active conformations. The crystal structures of α**_**M**_**γ**^**CIT**^**and α**_**M**_**β are shown as representatives.***A*, superposition of the α_M_γ^CIT^ (left panel) and α_M_β (right panel) structures onto the WT α^Mg^γ^Mg+CIT^ structure (PDB 5GRH) showing that the key structure elements (the α7 helices and β5-β6 loops) of the α_M_γ^CIT^ and α_M_β structures assume active conformations as those in the WT α^Mg^γ^Mg+CIT^ structure. For clarity, the WT α^Mg^γ^Mg+CIT^ structure is shown as semitransparent *gray* ribbon model. *B*, the heterodimer-heterodimer interfaces in the α_M_γ^CIT^ (left panel) and α_M_β (right panel) structures are mediated by the clasp domains. *C*, zoom-in views of the hydrogen-bonding networks at the heterodimer-heterodimer interfaces in the α_M_γ^CIT^ (left panel) and α_M_β (right panel) structures. PDB, Protein Data Bank.

Structural analyses show that the overall structures of the α_M_β and α_M_γ heterodimers bound with or without ligands all assume active conformations with the characteristic structural elements conforming to those in the active α^Mg^γ^Mg+CIT^ structure rather than the inactive α^Mg^γ structure. Specifically, the N-terminal regions of the α7 helices in the α, β, and γ subunits all adopt helical conformations, and the β5-β6 loops in all subunits are shifted toward the heterodimer (α-β and α-γ) interfaces (Figs. 4*A* and S7*A*). In addition, the key residues Tyr126^A^ at the active site and Tyr135^G^ at the allosteric site (or Tyr137^B^ at the pseudo-allosteric site) also adopt active conformations with their sidechains pointing toward the α-γ (or α-β) interface in favorable orientations for substrate (ICT) and activator (CIT) binding, respectively (Figs. 4*A* and S7*A*).

Like the WT αβ and αγ structures, all of the α_M_β and α_M_γ structures form heterotetramers and the heterodimer-heterodimer interfaces are mediated by the clasp domains. A detailed structural analysis shows that in the α_M_γ and α_M_γ^CIT^ structures, the hydrogen-bonding networks at the αγ-αγ interface are different from that in the α^Mg^γ structure but similar to that in the α^Mg^γ^Mg+CIT^ structure. Specifically, the α-Q139A mutation makes Ala139^A1^ unable to form hydrogen bonds with His131^A1^ and Ala139^A2^, and similarly, Ala139^A2^ cannot form hydrogen bonds with His131^A2^ and Ala139^A1^. As a result, the sidechain of His131^A1^ points toward the α-γ interface and forms a hydrogen bond with the sidechain of His140^G2^, and the sidechain of His140^G1^ forms hydrogen bonds with Glu148^G1^ and Glu148^G2^; and similarly, the sidechain of His131^A2^ and His140^G2^ makes equivalent hydrogen bonds (Figs 4, *B* and *C* and S7, *B* and *C*, left panels). The His131^A^-His140^G^ interactions render the β5-β6 loops to adopt active conformations, which lead the sidechains of Tyr126^A^ and Tyr135^G^ to adopt the active conformations as well.

In the apo α_M_γ structure, the β3^G^-α3^G^ and β5^G^-β6^G^ loops at the allosteric site are partially disordered, whereas in the α_M_γ^CIT^ structure, the β3^G^-α3^G^ and β5^G^-β6^G^ loops are well defined in the electron density map. The CIT binds to the allosteric site in a manner similar to that in the α^Mg^γ^Mg+CIT^ structure (Fig. S7*D*). The CIT binding apparently stabilizes the conformations of the β3^G^-α3^G^ and β5^G^-β6^G^ loops; however, it does not cause notable conformational changes of the αγ-αγ interface and the active site. Nevertheless, it is possible that the stabilization of the β3^G^-α3^G^ and β5^G^-β6^G^ loops by the CIT binding may further stabilize the active conformations of the αγ-αγ interface and the active site, which may explain the biochemical data showing that the α_M_γ heterodimer still exhibits a slight activation effect (2.0-fold) with the addition of activators, even though the activation effect is much weaker than that of the WT αγ heterodimer (20.8 fold) (Table 1).

In the WT αβ structures, the hydrogen-bonding network at the αβ-αβ interface is similar to that in the α^Mg^γ structure, indicative of inactive conformations (Figs. 3*C* and S6*B*). However, in all of the α_M_β structures, the hydrogen-bonding network at the αβ-αβ interface is similar to that in the α^Mg^γ^Mg+CIT^ structure or the α_M_γ structures, indicative of active conformations. In the WT αβ structures, the sidechain of Gln139^A1^ forms hydrogen bonds with the sidechains of Gln139^A2^ and His131^A1^. In addition, the sidechain of Glu150^B1^ (corresponding to Glu148^G1^) forms a hydrogen bond with the sidechain of His142^B2^ (corresponding to His140^G2^) (Fig. S6*B*). In the α_M_β structures, the α-Q139A mutation eliminates the Gln139^A1^-Gln139^A2^ hydrogen bond (Fig. 4, *B* and *C* and S7, *B* and *C*, right panels). The intramolecular hydrogen bonds His131^A1^-Gln139^A1^ and His131^A2^-Gln139^A2^ are replaced by the intermolecular hydrogen bonds His131^A1^-His142^B2^ and His131^A2^-His142^B1^, and in addition, His142^B1^ and His142^B2^ form extra hydrogen bonds with Glu150^B1^ and Glu150^B2^ (Fig. 4, *B* and *C* and S7, *B* and *C*, right panels). The changes of hydrogen-bonding interactions at the αβ-αβ interface induce conformational changes of the β5-β6 loops, which lead the sidechains of Tyr126^A^ and Tyr137^B^ to adopt the active conformations as well.

Taken together, our structural data demonstrate that the α-Q139A mutation alters the hydrogen-bonding networks at the αγ-αγ and αβ-αβ interfaces in a manner similar to that in the activation process of the WT αγ heterodimer upon the CIT binding to the allosteric site, which further induce conformational changes of the active site, the allosteric site, and the pseudo-allosteric site, leading them to assume active conformations similar to that in the α^Mg^γ^Mg+CIT^ structure. This provides the molecular basis for why the α_M_γ and α_M_β heterodimers are constitutively active (Table 1). Moreover, structural comparison shows that the intermolecular hydrogen-bonding interactions at the heterodimer-heterodimer interfaces in the (α_M_β)_2_ and (α_M_γ)_2_ structures are more extensive than those in the WT (αβ)_2_ and (αγ)_2_ structures. This provides the molecular basis for the SEC-MALS analysis and TSA assay results showing that α_M_β and α_M_γ always exist as heterotetramers at both low and high concentrations, and the α-Q139A mutation stabilizes the heterotetrameric forms of the α_M_β and α_M_γ heterodimers.

### The pseudo-allosteric site in the α_M_β heterodimer can bind allosteric activators

**Figure 5:**
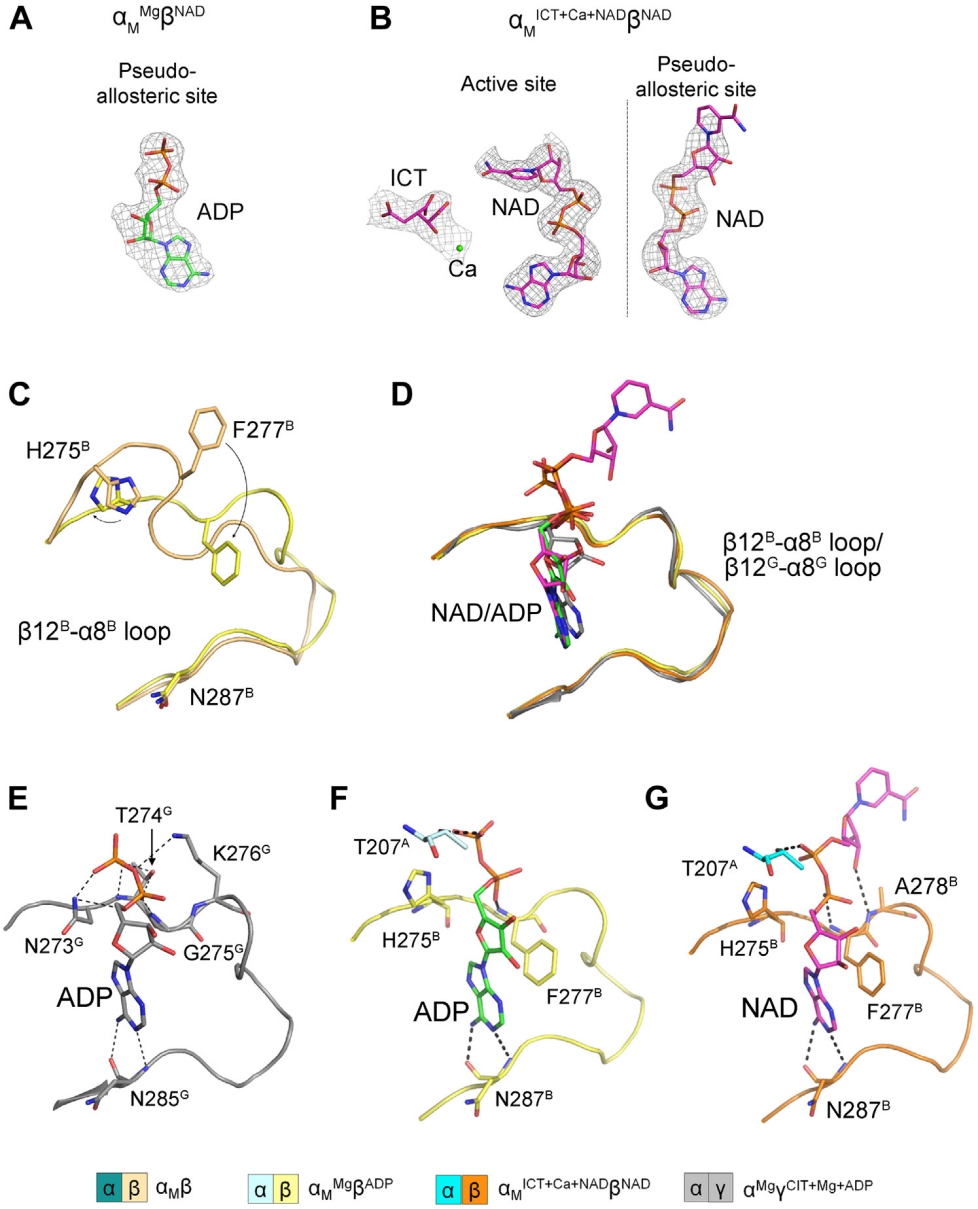
**Binding of ADP or NAD at the pseudo-allosteric site of α**_**M**_^**Mg**^**β**^**ADP**^**or α**_**M**_^**ICT+Ca+NAD**^**β**^**NAD**^**.***A*, composite omit map for ADP bound at the pseudo-allosteric site of α_M_^Mg^β^ADP^ (contour level = 1.0σ). *B*, composite omit map for ICT, NAD, and Ca^2+^ bound at the active site, and NAD bound at the pseudo-allosteric site of α_M_^ICT+Ca+NAD^β^NAD^ (contour level = 1.0σ). *C*, structural comparison of the β12^B^-α8^B^ loops at the pseudo-allosteric sites of α_M_β and α_M_^Mg^β^ADP^. *D*, structural comparison of the ADP/NAD binding to the (pseudo-)allosteric sites of α_M_^Mg^β^ADP^, α_M_^ICT+Ca+NAD^β^NAD^, and α^Mg^γ^CIT+Mg+ADP^. *E*, hydrogen-bonding interactions between ADP and the protein residues at the allosteric site of α^Mg^γ^CIT+Mg+ADP^. *F*, hydrogen-bonding interactions between NAD and the protein residues at the pseudo-allosteric site of α_M_^ICT+Ca+NAD^β^NAD^. *G*, hydrogen-bonding interactions between ADP and the protein residues at the pseudo-allosteric site of α_M_^Mg^β^ADP^. ICT, isocitrate.

Our previous biochemical and structural studies showed that the WT αβ heterodimer cannot be activated by the activators because the pseudo-allosteric site is spatially occupied by the β3^B^-α3^B^ and β12^B^-α8^B^ loops and thus cannot bind CIT and ADP. Intriguingly, in this study, we found that there is an ADP bound at the pseudo-allosteric site with clearly defined electron density in the α_M_^Mg^β^ADP^ structure (Fig. 5*A*), and there is an NAD bound at the pseudo-allosteric site albeit only the ADP-ribose moiety is well defined while the nicotinamide moiety is poor defined in the electron density map in the α_M_^ICT+Ca+NAD^β^NAD^ structure (Fig. 5, *B*). Moreover, the NAD bound at the pseudo-allosteric site adopts a conformation different from that of NAD bound at the active site but similar to that of ADP bound at the pseudo-allosteric site.

In the apo α_M_β structure, the conformations of the β3^B^-α3^B^ and β12^B^-α8^B^ loops are similar to those in the WT αβ heterodimer. The binding of ADP/NAD at the pseudo-allosteric site causes substantial conformational changes of the sidechains of His275^B^ and Phe277^B^, which induce the β12^B^-α8^B^ loop to adopt a conformation similar to that of the β12^G^-α8^G^ loop in the WT αγ heterodimer (Fig. 5, *C* and *D*). The binding mode of ADP/NAD at the pseudo-allosteric site is also similar to that of ADP at the allosteric site in the α^Mg^γ^CIT+Mg+ADP^ structure. In particular, in the α^Mg^γ^CIT+Mg+ADP^ structure, ADP forms hydrogen bonds with Asn273^G^, Thr274^G^, Lys276^G^, and Asn287^G^ in the β12^G^-α8^G^ loop (Fig. 5*E*). In the α_M_^Mg^β^ADP^ and α_M_^ICT+Ca+NAD^β^NAD^ structures, ADP or NAD form hydrogen bonds with His275^B^, Phe277^B^, Ala278^B^, and Asn287^B^ (corresponding to Asn273^G^, Gly275^G^, Lys276^G^, and Asn287^G^) in the β12^B^-α8^B^ loop and Thr207^A^ from the α small domain (Fig. 5, *F* and *G*). Besides, the sidechain of Phe277^B^ makes a π-π interaction with the adenine moiety of the ligand, which further stabilizes the ligand binding (Fig. 5, *F* and *G*).

These results indicate that the α-Q139A mutation not only induces conformational changes of the αβ-αβ interface and the active site, rendering the α_M_β mutant constitutively active, but also induces conformational changes of the pseudo-allosteric site enabling it to bind ADP/NAD. This observation is supported by our biolayer interferometry analysis results showing that while ADP has no detectable binding to the WT αβ heterodimer, it binds to the α_M_β heterodimer with a *K*_D_ of 522 ± 10 μM (Fig. S8). The ADP binding at the pseudo-allosteric site may in turn stabilize the active conformation of the αβ-αβ interface and the active site, which may explain our biochemical data showing that the addition of activators has a weak activation effect (1.7-fold) for the α_M_β heterodimer but not for the WT αβ heterodimer (Table 1).

### The α_M_^ICT+Ca+NAD^β^NAD^ structure represents a pseudo-Michaelis complex

**Figure 6:**
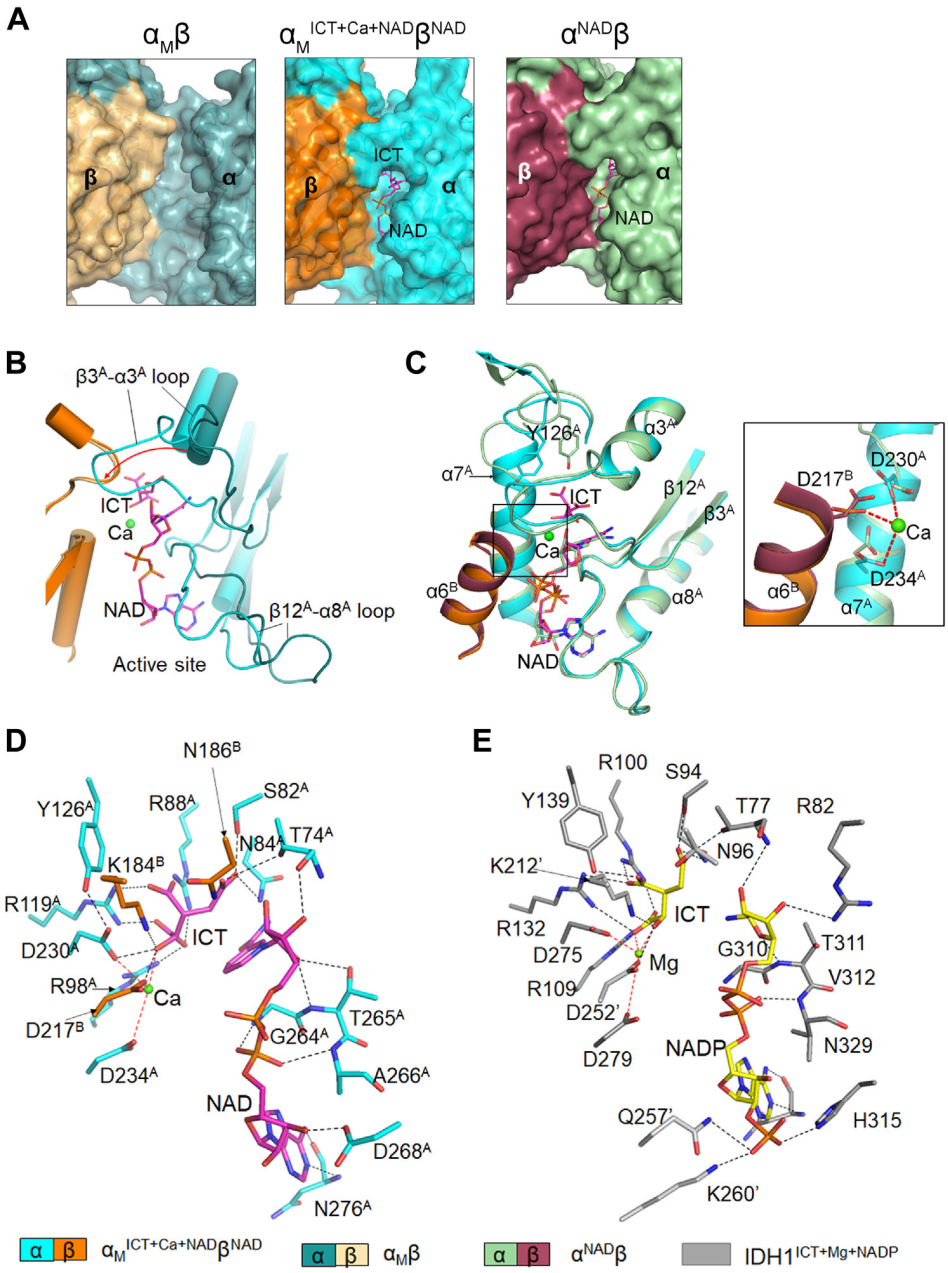
**Binding of ICT, Ca**^**2+**^**, and NAD at the active site of α**_**M**_^**ICT+Ca+NAD**^**β**^**NAD**^**.***A*, surface representation of the active sites of α_M_β (open conformation), α_M_^ICT+Ca+NAD^β^NAD^ (closed conformation), and α^NAD^β (PDB 6KDY; closed conformation). *B*, comparison of the active sites of α_M_β and α_M_^ICT+Ca+NAD^β^NAD^. *C*, comparison of the active sites of α_M_^ICT+Ca+NAD^β^NAD^ and α^NAD^β. The zoom-in view shows the structure of the metal-binding site. The coordination bonds between the Ca^2+^ ion and the protein residues are shown in *red lines*. *D*, interactions between ICT, Ca^2+^, NAD, and the protein residues at the active site of α_M_^ICT+Ca+NAD^β^NAD^. Hydrogen bonds are indicated with *black dash lines* and coordination bonds are indicated with *red dash lines*. *E*, interactions between ICT, Ca^2+^, NADP, and the protein residues at the active site of human IDH1^ICT+Ca+NADP^ homodimer (PDB 6PAY). Residues from the other subunit in the IDH1 homodimer are indicated with prime. ICT, isocitrate; PDB, Protein Data Bank.

In the α_M_^ICT+Ca+NAD^β^NAD^ structure, the active site is bound with the substrate ICT, the cofactor NAD, and a Ca^2+^ ion. This is the first pseudo-Michaelis complex reported for HsIDH3 and other NAD-IDHs. In our previous studies, various attempts to cocrystallize WT αβ and αγ in the presence of ICT, NAD, and metal ions failed to yield a pseudo-Michaelis complex. Structural comparison shows that in all of the WT and mutant αβ and αγ structures without NAD or ICT bound at the active site, the α subunit always assumes an open conformation; in contrast, in the α_M_^ICT+Ca+NAD^β^NAD^ and α^NAD^β (PDB 6KDY) structures, the α subunit assumes a closed conformation (Fig. 6*A*). The binding of NAD to the active site induces the α large domain and in particular, the β3^A^-α3^A^ and β12^A^-α8^A^ loops to rotate toward the α-β interface, resulting in a closed conformation of the active site. Consequently, the β3^A^-α3^A^ and β12^A^-α8^A^ loops partially cover the active site and are in proper positions to interact with NAD and ICT (Fig. 6*B*). Additional binding of ICT does not induce further conformational changes of the active site.

Although both WT αβ and mutant α_M_β heterodimers can bind NAD at the active site, our biochemical data show that the WT αβ heterodimer has only a basal activity, whereas the α_M_β heterodimer is constitutively active. In the α^NAD^β structure, the N-terminal regions of the α7 helices adopt the inactive loop conformations and the sidechain of Tyr126^A^ adopts an inactive conformation as well, and consequently, the sidechains of Asp230^A^, Asp234^A^, and Asp217^B^ are not in proper conformations to bind the metal ion (Fig. 6*C*). In contrast, in the α_M_^ICT+Ca+NAD^β^NAD^ structure, the N-terminal regions of the α7 helices adopt the active helical conformations and the sidechain of Tyr126^A^ also adopts the active conformation, which lead the sidechains of Asp230^A^, Asp234^A^, and Asp217^B^ in proper orientations to form coordination bonds with the Ca^2+^ ion (Fig. 6*C*). In addition, the sidechain of Asp230^A^ is in close proximity to the hydroxyl group of Tyr126^A^ (2.8 Å) and the ε-ammonium group of Lys184^B^ (2.9 Å). Thus, the Lys184^B^-Asp230^A^-Tyr126^A^ triad appears to be in favorable geometries for proton transfer (Fig. 6*D*). These results indicate that the active site in α_M_^ICT+Ca+NAD^β^NAD^ is in the active conformation to exert the catalytic activity, whereas the active site in α^NAD^β is in the inactive conformation unable to exert the catalytic reaction. This explains why the WT αβ and mutant α_M_β have distinct enzymatic activity.

As the α_M_^ICT+Ca+NAD^β^NAD^ structure represents the pseudo-Michaelis complex of the α_M_β heterodimer in the active state, it allows us to identify the key residues involved in the recognition and binding of the cofactor NAD, the substrate ICT, and the metal ion. The NAD is bound at the deep pocket of the active site with the nicotinamide moiety laying close to the ICT-binding site and forming a hydrogen bond with the γ-carboxyl group of ICT and the adenine moiety at the far end of the active site (Fig. 6*D*). The residues involved in the NAD binding, including Thr74^A^, Asn84^A^, Gly264^A^, Thr265^A^, Asp268^A^, Asn276^A^, and Asn186^B^, are highly conserved in eukaryotic NAD-IDHs (Fig. S9). Notably, the sidechain of Asp268^A^ makes a hydrogen-bonding interaction with the ribose 2′-OH of the adenosine moiety, which distinguishes NAD from NADP, consistent with the notion that Asp268^A^ is critical for cofactor specificity (Fig. 6, *D* and *E*) (20). The ICT makes extensive hydrogen-bonding interactions with the surrounding residues (Fig. 6*D*). Specifically, the α-carboxyl group interacts with Arg88^A^, Arg98^A^, and Arg119^A^; the β-carboxyl group interacts with Arg88^A^ and Arg119^A^; the γ-carboxyl group interacts with Thr74^A^, Ser82^A^, Asn84^A^; and the α-hydroxyl group forms hydrogen bonds with Lys184^B^, Asp217^B^, and Asp230^A^, and a coordination bond with the Ca^2+^ ion. Residues Asp230^A^, Asp234^A^, and Asp217^B^ as well as the ICT are responsible for the proper binding and positioning of the metal ion.

**Figure 7:**
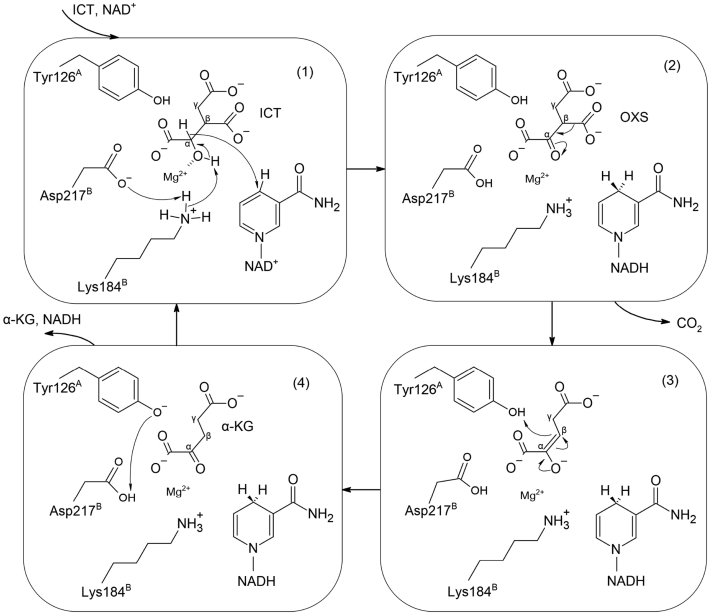
**Proposed catalytic mechanism for HsIDH3.** (1) NAD reduction (2). Decarboxylation on the C_β_ atom and double bond rearrangement (3). Protonation on the C_β_ atom and release of CO_2_ (4). Release of the product. Catalytic residues are numbered according to the αβ heterodimer of HsIDH3.

Structural comparison of α_M_^ICT+Ca+NAD^β^NAD^ and the previously reported pseudo-Michaelis complex of HsIDH1 (PDB 6PAY) shows that ICT, NAD (P), and metal ion bind to the active sites of HsIDH1 and HsIDH3 in a similar manner (Fig. 6, *D* and *E*). Despite the low sequence identity between human NAD-IDH and human NADP-IDHs (19.2% between HsIDH3α and HsIDH1 and 16.7% between HsIDH3α and HsIDH2), the critical amino acids involved in the binding of substrate and metal ion and the catalytic reaction are well conserved in both NAD- and NADP-IDHs (Fig. 6, *D* and *E*). Thus, based on the catalytic mechanism of human NADP-IDHs (24, 25), we can propose the catalytic mechanism for HsIDH3 and other mammalian NAD-IDHs (Fig. 7). Specifically, the α-hydroxyl group of ICT forms a coordination bond with the metal ion, which polarizes the α-hydroxyl group of ICT. Then, the sidechain of Asp230^A^ deprotonates the sidechain of Lys184^B^ and the latter acts as a general base to deprotonate the α-hydroxyl group of ICT. The hydride ion is expulsed from the C_α_ atom of ICT and accepted by NAD. This step results in formation of the unstable intermediate product oxalosuccinate, which is quickly decarboxylated on the C_β_ atom, making carbon dioxide to separate from oxalosuccinate. The C_β_ atom is subsequently protonated by the sidechain of Tyr126^A^ to form the product α-KG. Finally, Tyr126^A^ accepts a proton from Asp230^A^ to return back to the initial protonation state. In this proposed mechanism, the Lys184^B^-Asp230^A^-Tyr126^A^ triad is the key residues responsible for the catalytic reaction. It is also clearly shown that the active site consists of residues from both α and β (or γ) subunits of the heterodimers, which are involved in the binding of the substrate and the metal ion and in the catalytic reaction, and the α subunit alone is insufficient to carry out the catalytic reaction (Fig. 7).

### Structure of the unliganded (α_M_βα_M_γ)_2_ holoenzyme assumes an active conformation

Crystallization of the WT HsIDH3 holoenzyme in the presence of activators (CIT or/and ADP) yielded crystals at several conditions; however, these crystals diffracted X-rays to only low-resolution (about 6–7 Å), prohibiting us from obtaining the crystal structure of the holoenzyme in an active form with CIT and/or ADP bound at the allosteric site and/or pseudo-allosteric site. On the other hand, our biochemical and structural studies show that the α-Q139A mutation renders the α_M_γ and α_M_β heterodimers constitutively active, and the structures of the α_M_γ and α_M_β heterodimers in both absence and presence of the activators assume active conformations similar to that of the α^Mg^γ^CIT^ heterodimer. Thus, we tried to crystallize the (α_M_βα_M_γ)_2_ holoenzyme in the absence of activators and finally were able to solve the crystal structure of the mutant holoenzyme with an active conformation at 4.5 Å resolution (Table 2). The structure of the (α_M_βα_M_γ)_2_ holoenzyme belongs to space group *C*222_1_ and each asymmetric unit contains two (α_M_βα_M_γ)_2_ heterooctamers. Most residues of the polypeptide chains of the α, β, and γ subunits are defined with good electron density.

The overall structure of the (α_M_βα_M_γ)_2_ holoenzyme is similar to that of the WT (αβαγ)_2_ holoenzyme (Fig. S10*A*). Intriguingly, when one heterotetramer of the two structures is superposed, the other heterotetramer of the mutant holoenzyme shows approximately 20° rotation relative to that of the WT holoenzyme (Fig. S10*B*). The biological implication of this conformational change is unclear. Moreover, in the mutant (α_M_βα_M_γ)_2_ structure, the active site and the allosteric site of the αγ heterodimer, the active site and the pseudo-allosteric site of the αβ heterodimer, and the αβ-αγ interface appear to assume active conformations. In particular, the N-terminal regions of the α7 helices in all of the α, β, and γ subunits assume helical conformations similar to that in the active α^Mg^γ^Mg+CIT^ structure (Fig. S10*C*). In addition, the sidechains of Tyr126^A^ and Tyr137^B^ in the α_M_β heterodimer adopt active conformations; and while the sidechain of Tyr136^G^ in the α_M_γ heterodimer is not clearly defined in the electron density map, the sidechain of Tyr126^A^ also adopts active conformation (Fig. S10*D*). In other words, the α-Q139A mutation induces conformational changes of the clasp domains of the α subunits and the αβ-αγ interface and subsequently the active sites, the allosteric sites, and the pseudo-allosteric sites, which mimic the conformational changes induced by the binding of activators at the allosteric site, rendering the mutant holoenzyme to adopt the active conformation. Nevertheless, as the resolution of the (α_M_βα_M_γ)_2_ structure is low, we could not perform detailed structural analysis of the αβ-αγ interface and compare it with that in the inactive WT (αβαγ)_2_ structure.

## Discussion

Human NAD-IDH or HsIDH3 catalyzes the conversion of ICT to α-KG in the tricarboxylic acid cycle. The composing α, β, and γ subunits of HsIDH3 play distinct roles in the assembly, catalytic reaction, and allosteric regulation of the holoenzyme. Mutations and dysregulation of different subunits of HsIDH3 have been associated with various diseases including cancers (26, 27, 28). Therefore, the functional, structural, and mechanistic studies of HsIDH3 have important biological and biomedical significances. Our previous biochemical data showed that the WT HsIDH3 holoenzyme exhibits a notably higher activity than the sum of the activities of the composing αβ and αγ heterodimers both in the absence and presence of activators (CIT and ADP) (18). The mutant HsIDH3 holoenzyme containing the α-Y126F mutation at the active site in either αβ or αγ heterodimer exhibits about 50% of the activity of the WT HsIDH3 holoenzyme and retains the activation effect by the activators; but, the mutant HsIDH3 holoenzyme containing the α-Y126F mutation in both heterodimers completely abolishes the activity (18). These results indicate that in the HsIDH3 holoenzyme, both αβ and αγ heterodimers have catalytic activity and can be activated by the activators and the binding of activators to the allosteric site in the γ subunit can regulate the α subunit in both heterodimers. However, the molecular mechanism of the allosteric activation of the HsIDH3 holoenzyme remains elusive, and in particular, how the activation signal is transmitted from the γ subunit to the α subunit in both αβ and αγ heterodimers remains elusive.

Previous structural and biochemical studies of the αγ heterodimer demonstrated that upon binding of CIT or/and ADP to the allosteric site, the activation signal is transmitted from the γ subunit to the α subunit through conformational changes of several structural elements, leading to the remodeling of a series of hydrogen bonds at the α-γ and the αγ-αγ interfaces (19). In particular, at the αγ-αγ interface, the Gln139^A1^-Gln139^A2^ hydrogen bond is disrupted and the intramolecular hydrogen bond Gln139^A^-His131^A^ is replaced by an intermolecular hydrogen bond His131^A1^-His140^G2^. In this study, we found that the α_M_γ heterodimer is constitutively active regardless of the absence and presence of activators. In the α_M_γ structures, the hydrogen-bonding network at the αγ-αγ interface is similar to that in the active α^Mg^γ^Mg+CIT^ structure. In other words, the α-Q139A mutation causes rearrangement of the hydrogen-bonding network at the αγ-αγ interface to mimic the activation process of the αγ heterodimer upon the binding of activators, which induces the conformational changes of the active site to assume the active conformation, rendering the α_M_γ heterodimer constitutively active. These results also indicate that the rearrangement of hydrogen-bonding network involving Gln139^A^ at the αγ-αγ interface plays a critical role in the transmission of the activation signal from the γ subunit to the α subunit. In the α_M_γ structures, the allosteric site retains the ability to bind CIT (and very likely ADP), and the CIT binding does not induce additional conformational changes of the active site and the αγ-αγ interface but may further stabilize the active conformations of the αγ-αγ interface and the active site and thus achieves a weak activation effect.

The structural and biochemical studies of the αβ heterodimer demonstrated that the αβ heterodimer alone has only basal activity and cannot be activated by CIT or ADP. The β subunit contains a pseudo-allosteric site, which is spatially occupied in part by the β3^B^-α3^B^ and β12^B^-α8^B^ loops and hence cannot bind the activators, leading to the suggestion that the β subunit plays only a structural role but no regulatory role (18). In this study, we found that the α-Q139A mutation renders the α_M_β mutant constitutively active. We further determined the crystal structure of a pseudo-Michaelis complex α_M_^ICT+Ca+NAD^β^NAD^, which confirms that the active site of the αβ heterodimer can bind the substrate and cofactor. In the α_M_β structures, the α-Q139A mutation also causes rearrangement of the hydrogen-bonding network at the αβ-αβ interface and induces conformational changes of the active site to assume the active conformation in a similar manner as that in the α_M_γ heterodimer. Moreover, the conformational changes at the αβ-αβ interface also cause conformational changes of the pseudo-allosteric site, enabling it to bind ADP or NAD in a similar manner as the allosteric site in the γ subunit to bind ADP. These results suggest that the αβ-αβ interface could transmit the activation signal to the α subunit in the αβ heterodimer.

In this work, we further found that the WT αβ and αγ heterodimers form dimers of heterodimers or heterotetramers [(αβ)_2_ and (αγ)_2_] in the crystal structures, and the heterodimer-heterodimer (αβ-αβ and αγ-αγ) interfaces are mediated by their clasp domains in a similar way as the assembly of the αβ and αγ heterodimers into the αβαγ heterotetramer in the unliganded HsIDH3 holoenzyme. In particular, the hydrogen-bonding networks at the αγ-αγ interface in the inactive α^Mg^γ structure and the αβ-αβ interface in the inactive α^NAD^β structure are formed in the same manner as that at the αβ-αγ interface in the inactive (αβαγ)_2_ structure. Furthermore, our moderate-resolution structure of the unliganded (α_M_βα_M_γ)_2_ holoenzyme shows that like in the mutant α_M_β and α_M_γ structures, the α-Q139A mutation induces the αβ-αγ interface as well as both α_M_β and α_M_γ heterodimers in the mutant holoenzyme to assume active conformations. These results indicate that the (αβ)_2_ and (αγ)_2_ heterotetramers mimic the αβαγ heterotetramer and the rearrangement of hydrogen-bonding network at the heterodimer-heterodimer interface in the activation of the αβ and αγ heterodimers mimics that at the αβ-αγ interface in the activation of the αβαγ heterotetramer. There is structural communication between the αβ and αγ heterodimers in the αβαγ heterotetramer through the clasp domains. Specifically, upon the CIT binding to the allosteric site, the activation signal (the conformational changes at the allosteric site) is transmitted to the active sites of both αβ and αγ heterodimers through the rearrangement of hydrogen-bonding network at the heterodimer-heterodimer interface.

**Figure 8:**
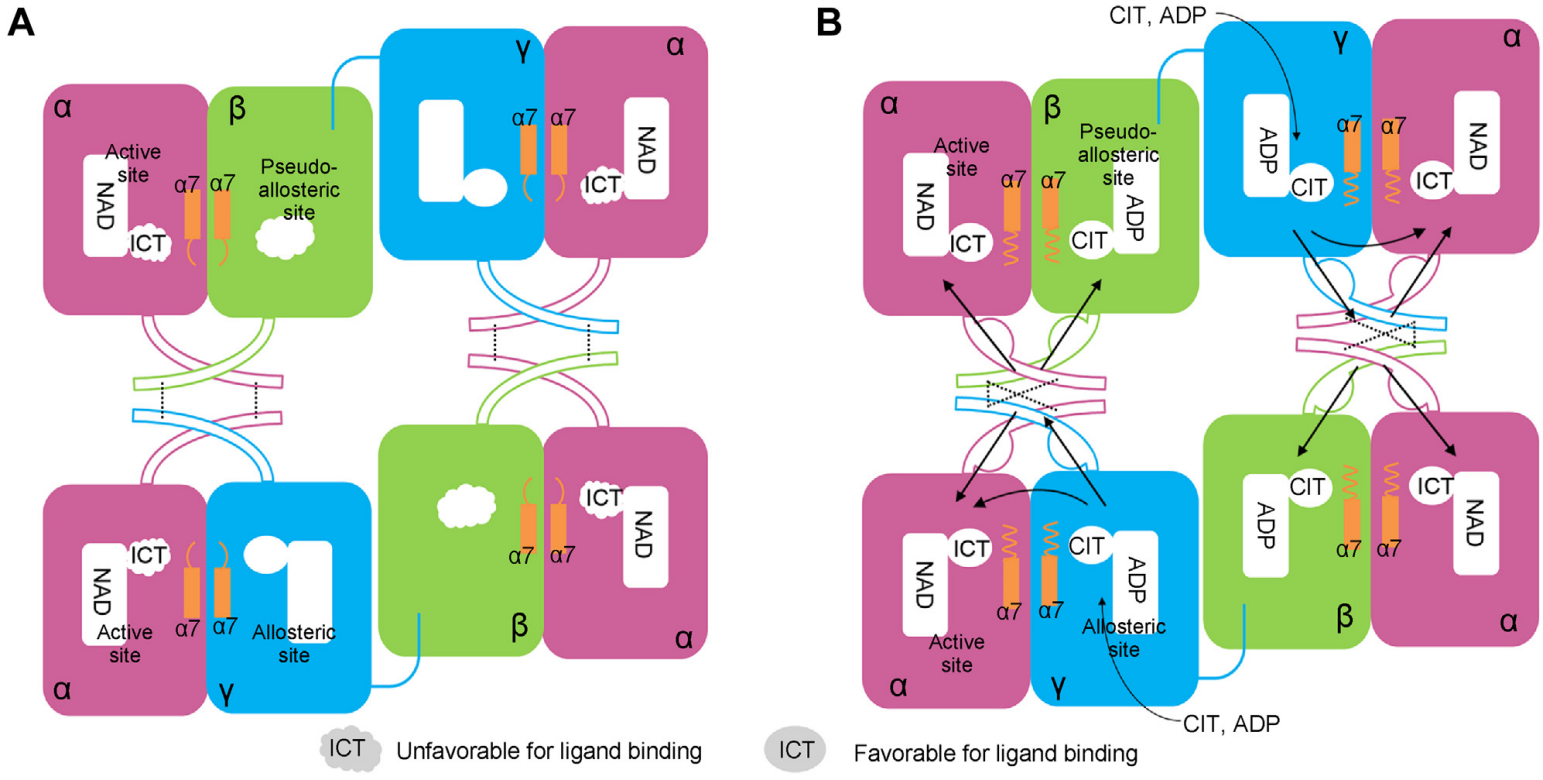
**Proposed molecular mechanism for the allosteric activation of HsIDH3 holoenzyme.***A*, inactive state. The active sites of both αβ and αγ heterodimers can bind the substrate ICT with low affinity. The pseudo-allosteric site of the β subunit adopts a conformation unfavorable for activator binding. *B*, active state. The binding of activators (CIT or/and ADP) to the allosteric site in the γ subunit induces the rearrangement of the hydrogen-bonding network at the α-γ interface and the αγ-αβ interface and subsequently induces the conformational changes of the active sites in both αβ and αγ heterodimers to adopt the active conformation. The transmission of the activation signal between subunits is indicated by *arrows*. The critical hydrogen bonds in the heterodimer-heterodimer interface involved in the activation signal transmission are indicated by dash lines. CIT, citrate; ICT, isocitrate.

Based on these biochemical and structural data, we can propose the molecular mechanism for the allosteric activation of the HsIDH3 holoenzyme (Fig. 8). At the inactive state, the active sites of both αβ and αγ heterodimers can bind substrate ICT with low affinity and exert low basal activities and thus the HsIDH3 holoenzyme exhibits a low basal activity (Fig. 8*A*). The binding of activators (CIT or/and ADP) to the allosteric site in the γ subunit induces the conformational changes of the allosteric site, which causes the rearrangement of hydrogen-bonding network at the α-γ interface and the αγ-αβ interface (Fig. 8*B*). Specifically, at the αγ-αβ interface, the hydrogen bonds including Gln139^A1^-Gln139^A2^, Gln139^A1^-His131^A1^, and Gln139^A2^-His131^A2^ (residues of the α subunit of the αβ heterodimer and the α subunits of the αγ heterodimer are superscripted by “A1” and A2”, respectively) are disrupted, and new hydrogen bonds including His131^A1^-His140^G^, His131^A2^-His142^B^, His142^B^-Glu148^G^, and His140^G^-Glu150^B^ are formed, resulting in a more extensive intermolecular hydrogen-bonding interactions. These changes induce the conformational changes of the active sites in both αβ and αγ heterodimers to adopt the active conformations, leading both heterodimers to exert high catalytic activity (Fig. 8*B*). In addition, these changes also induce the conformational changes of the pseudo-allosteric site in the β subunit enabling it to bind the activators, which further stabilizes the active conformations of the αβ-αγ interface and the active sites to enhance the allosteric activation effect. These effects together achieve the synergistic functions of the αβ and αγ heterodimers in the holoenzyme and thus the optimal activity of the holoenzyme.

## Experimental procedures

### Cloning, expression, and purification

The WT and α-Q139A-containing αβ and αγ heterodimers and (αβαγ)_2_ heterooctamer of HsIDH3 were prepared as described previously (19, 21, 23). Briefly, the DNA fragments encoding the α, β, and γ subunits of HsIDH3 were cloned into the coexpression vector pQlinkN with the C-terminals of the β and γ subunits attached with a TEV protease cleavage site and a His_6_ tag, yielding the pQlinkN-α-β-tev-His_6_ and pQlinkN-α-γ-tev-His_6_ plasmids. The plasmids were transformed into *Escherichia coli* BL21 (DE3) Codon-Plus strain (Novagen). When the culture of the transformed cells reached an *A*_600_ of 0.5, the protein expression was induced by 0.4 mM IPTG for 20 h at 24 °C. The cells were harvested and then sonicated on ice in the lysis buffer (50 mM Hepes, pH 7.4, 200 mM NaCl, 10% glycerol, and 7.2 mM β-ME) supplemented with 1 mM PMSF. The target proteins were purified by affinity chromatography using a Ni-NTA column (Qiagen), with the lysis buffer supplemented with 20 mM and 200 mM imidazole serving as the washing buffer and elution buffer, respectively. The elution fraction was dialyzed overnight against the lysis buffer supplemented with TEV protease to cleave the His_6_-tag off the target protein. The cleavage mixture was reloaded on a Ni-NTA column and washed with the lysis buffer supplemented with 10 mM imidazole. The flow-through fraction containing the target protein was further purified by gel filtration using a Superdex 200 10/60 GL column (GE Healthcare) equilibrated with the storage buffer (10 mM Hepes, pH 7.4, 200 mM NaCl, and 5 mM β-ME). The (αβαγ)_2_ heterooctamer was prepared by copurifying the separately expressed αβ and αγ heterodimers using the same methods as for the αβ and αγ heterodimers. The purities of the proteins were analyzed by 12% SDS-PAGE with Coomassie blue staining. The mutant αβ and αγ heterodimers containing point mutations were constructed using the QuikChange Site-Directed Mutagenesis kit (StrateGene). Expression and purification of the mutant αβ and αγ heterodimers and (αβαγ)_2_ heterooctamer of HsIDH3 were carried out using the same methods as for the WT proteins.

### SEC-MALS analysis

The purities and molecular weights of the proteins were analyzed by a SEC-MALS instrument consisting of an Agilent 1260 Infinity Isocratic Liquid Chromatography System, a Wyatt Dawn Heleos II Multi-Angle Light Scattering Detector, and a Wyatt Optilab T-rEX Refractive Index Detector (Wyatt Technology). Analytical SEC was performed at 24 °C using a Superdex 200 10/300 Gl column (GE Healthcare) equilibrated with a mobile phase containing 10 mM Hepes (pH 7.4), 200 mM NaCl, and 5 mM β-ME. Hundred microliters protein solution was injected into the column and eluted at a flow rate of 0.4 ml/min. The column effluent was monitored simultaneously with three detectors for UV absorption, light scattering, and refractive index. Light scattering detector is used for molecular weight measurement; UV and refractive index detectors are used for concentration measurements in two orthogonal ways. The data were analyzed using the ASTRA software (Wyatt Technology) to determine the molecular weight of the protein (29).

### Crystallization, diffraction data collection, and structure determination

Crystallization was performed using the hanging drop vapor diffusion method at 20 °C by mixing equal volume of protein solution (10 mg/ml) and reservoir solution. Crystals of α_M_β in apo form grew in crystallization drops containing 8% (v/v) Tacsimate (pH 7.0) and 20% (w/v) PEG 3350. Crystals of α_M_β in complex with ICT, NAD, and Ca^2+^ (α_M_^ICT+Ca+NAD^β^NAD^) grew in drops containing 0.2 M sodium malonate (pH 6.0), 0.1 M calcium acetate, 20% (w/v) PEG 3350, 40 mM ICT, 40 mM NAD, and 5 mM CaCl_2_. Crystals of α_M_^Mg^β^ADP^ grew in drops containing 0.1 M sodium cacodylate (pH 5.1), 10% (w/v) PEG 8000, 4 mM ADP, 4 mM CIT, and 0.4 mM MgCl_2_. Crystals of α_M_γ in apo form grew in drops containing 0.1 M Mes (pH 6.0) and 20% (w/v) PEG MME 2000. Crystals of α_M_γ in complex with CIT (α_M_γ^CIT^) grew in drops containing 0.02 M citric acid, 0.08 M Bis-tris propane (pH 8.8), and 16% (w/v) PEG 3350. Crystals of the (α_M_βα_M_γ)_2_ holoenzyme grew in drops containing 0.2 M NaCl, 0.1 M Mes (pH 6.5), and 10% (w/v) PEG 4000. The crystals were cryoprotected using the reservoir solutions supplemented with 25% ethylene glycol prior to diffraction data collection. Diffraction data were collected at 100 K at Shanghai Synchrotron Radiation Facility and processed with HKL2000 (30). Statistics of the diffraction data are summarized in Table 2.

All structures of the α_M_β and α_M_γ heterodimers were solved with the molecular replacement method as implemented in program Phenix (31) using the structure of the αβ heterodimer (PDB 6KDF) or the α^Mg^γ heterodimer (PDB 5GRH) as the search model. The structure of the (α_M_βα_M_γ)_2_ holoenzyme was solved with the molecular replacement method using the structure of the apo HsIDH3 holoenzyme (PDB 7CE3) as the search model. Initial structure refinement was carried out with program Phenix (31) and final structure refinement was performed with program REFMAC5 (32). Model building was performed with program Coot (33). Stereochemistry and quality of the structure models were analyzed using programs in the CCP4 suite (34). Structure figures were prepared using PyMol (35). Statistics of the structure refinement and the final structure models are also summarized in Table 2.

### Enzymatic activity assay

Enzymatic activities of the WT and mutant αβ and αγ heterodimers and (αβαγ)_2_ heterooctamer of HsIDH3 were determined using the method as described previously (19, 21). The standard reaction solution (1 mL) consisted of 2 ng/ml enzyme, 33 mM Tris-acetate (pH 7.4), 40 mM ICT, 2 mM MgCl_2_, and 3.2 mM NAD. The activity is defined as the micromoles of NADH produced per minute per milligram of enzyme (μmol∙min^−1^ mg^−1^). The kinetic data were measured in the absence or presence of activators (1 mM CIT and 1 mM ADP) with varied concentrations of ICT (0–40 mM) to obtain the *V*_max_ and *S*_0.5_ for ICT. The kinetic parameters were obtained by fitting the kinetic data into the nonlinear Michaelis–Menten equation “*V* = *V*_max_∗[S]^ˆ^h/(*S*_0.5_^ˆ^h+[S]^ˆ^h)” using program GraphPad Prism (GraphPad Software), where “[S]” is the concentration of ICT, “*V*_max_” is the maximal velocity, and “*S*_0.5_” is the apparent *K*_m_ (the Michaelis constant). All experiments were performed in triplicates and the values were the averages of the measurements with the standard errors. Statistical analysis was performed by unpaired Student’s *t* test using GraphPad.

### TSA

Protein stability of the WT and mutant HsIDH3 were analyzed by TSA using a Prometheus NT48 instrument (Nanotemper Technologies). The protein samples were prepared in 10 mM Hepes (pH 7.4) and 150 mM NaCl to a final concentration of 1 mg/ml and loaded into the standard capillaries. The temperature gradient was performed in the range of 25 to 90 °C with a ramp of 1.5 °C/min. Fluorescence intensities were monitored at wavelengths of 330 nm and 350 nm, and the fraction of unfolded protein was calculated by the fluorescence ratio of 350/330 nm. All experiments were performed twice and the values were the averages of the measurements with the standard errors. Statistical analysis was performed by unpaired Student’s *t* test using GraphPad Prism.

### Biolayer interferometry assay

The binding of ADP with the WT or mutant αβ heterodimer was measured using an Octet RED96 instrument (ForteBio Corp). All experiments were carried out at 25 °C in a buffer consisting of 10 mM Hepes (pH 7.4), 150 mM NaCl, and 0.05% (v/v) Tween-20. The protein was incubated with biotin at a 1:1 M ratio at room temperature for 30 min for biotinylation. Then, the biotinylated protein was loaded onto streptavidin biosensors (ForteBio Corp) at 100 μg/ml for 600 s to reach a response of approximately 0.6 nm. The binding of ADP was measured at a concentration range of 62.5 μM to 1 mM. Both association and dissociation were allowed to last for 600 s. Nonspecific binding were subtracted by using sensors saturated with buffer alone.

## Data availability

The crystal structures of α_M_β, α_M_^ICT+Ca+NAD^β^NAD^, α_M_^Mg^β^ADP^, α_M_γ, α_M_γ^CIT^, and α_M_βα_M_γ have been deposited in the Protein Data Bank with accession codes 8GRB, 8GRU, 8GRD, 8GRG, 8GRH, and 8GS5, respectively. All remaining data are contained within the article.

## Supporting information

This article contains supporting information.

## Conflict of interest

The authors declare that they have no conflicts of interest with the contents of this article.
