# Supplementary material for: Structures of a constitutively active mutant of human IDH3 reveal new insights into the mechanisms of allosteric activation and the catalytic reaction
Source: J Biol Chem. 2022 Nov 12;298(12):102695. doi: 10.1016/j.jbc.2022.102695 (PMC9731866; doi:10.1016/j.jbc.2022.102695)
Supplement: Supplemental Figures S1–S10 [file mmc1.pdf]

Supporting information for:

Structures of a constitutively active mutant of human IDH3 reveal new insights into the mechanisms of allosteric activation and the catalytic reaction

Xingchen Chen[1,$], Pengkai Sun[1,$], Yan Liu[1,2], Senlin Shen[1,3], Tengfei Ma[1], and Jianping Ding[1,2,4,*]

[1] State Key Laboratory of Molecular Biology, Shanghai Institute of Biochemistry and Cell Biology, Center for Excellence in Molecular Cell Science, University of Chinese Academy of Sciences, Chinese Academy of Sciences, 320 Yueyang Road, Shanghai 200031, China

[2] School of Life Science and Technology, ShanghaiTech University, 393 Huaxia Zhong Road, Shanghai 201210, China

[3] School of Life Sciences, Shanghai University, 333 Nanchen Road, Shanghai 200444, China

[4] School of Life Science, Hangzhou Institute for Advanced Study, University of Chinese Academy of Sciences, 1 Xiangshan Road, Hangzhou 310024, China

[$] These authors contributed equally to this work.

* To whom correspondence should be addressed: Jianping Ding, Email: jpding@sibcb.ac.cn; Tel: 86-21-5492-1619.

Contents

Figure S1. The composition and assembly of the HsIDH3 holoenzyme. S-2
Figure S2. Catalytic properties of the WT and mutant HsIDH3. .... S-3
Figure S3. Thermal shift assay (TSA) of $\alpha\beta$, $\alpha\gamma$, $\alpha_M\beta$ and $\alpha_M\gamma$. S-4
Figure S4. Crystal packing analysis of the $\alpha_M\gamma$ structure reveals that the γ large domain is involved in close contacts with the a large domain of a symmetry-related heterodimer. S-5
Figure S5. Structure of $\alpha^{NAD}\beta$ assumes inactive conformation. S-6
Figure S6. The heterodimer-heterodimer interfaces are similar in the structures of the inactive $\alpha\beta$ and $\alpha\gamma$ heterodimers and $(\alpha\beta\alpha\gamma)_2$ holoenzyme. S-7
Figure S7. The overall structures of $\alpha_M\gamma$ and $\alpha_M\beta$ assume active conformations in both apo and ligand-bound forms. S-8
Figure S8. Bio-layer interferometry (BLI) analysis of ADP binding to $\alpha\beta$ or $\alpha_M\beta$. S-9
Figure S9. Comparison of HsIDH3 with the homologous NAD-IDH from *Bos taurus* (BtIDH3), *Xenopus laevis* (XlIDH3), *Danio rerio* (DrIDH3) and *Saccharomyces cerevisiae* (ScIDH). S-10
Figure S10. Structure of the unliganded $(\alpha_M\beta\alpha_M\gamma)_2$ holoenzyme assumes active conformation. S-11

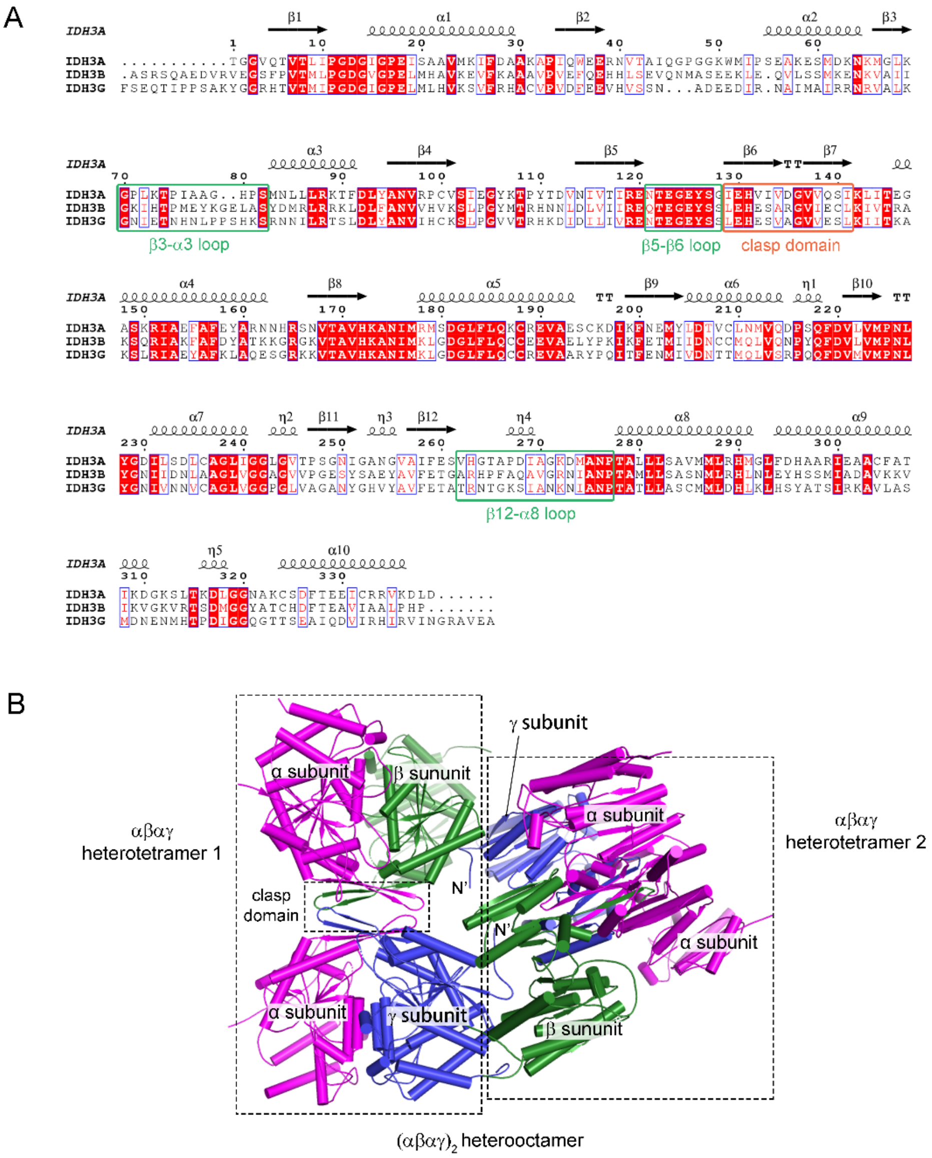

Figure S1. The composition and assembly of the HsIDH3 holoenzyme. (A) Sequence alignment of the α (IDH3A), β (IDH3B) and γ (IDH3G) subunits of HsIDH3. Secondary structure elements are labeled according to the α subunit in the $\alpha^{Mg}\gamma$ structure (PDB 5GRH). The loops involved in the ligand binding at the active site and the (pseudo) allosteric site are indicated by green boxes. The clasp domains are indicated by orange box. (B) The assembly of the $(\alpha\beta\alpha\gamma)_2$ heterooctamer of HsIDH3 (PDB 7CE3). The α (magenta), β (green) and γ (blue) subunits form the αβ and αγ heterodimers. The αβ and αγ heterodimers assemble into the αβαγ heterotetramer via the clasp domains, and two heterotetramers further assemble into a heterooctamer with the N-terminus of the γ subunit of one heterotetramer inserted into the β subunit of the other heterotetramer. The two heterotetramers are arranged in perpendicular to each other with a tetrahedron architecture; the two β and two γ subunits are arranged alternately to form the inner core, and the four α subunits are positioned on the periphery.

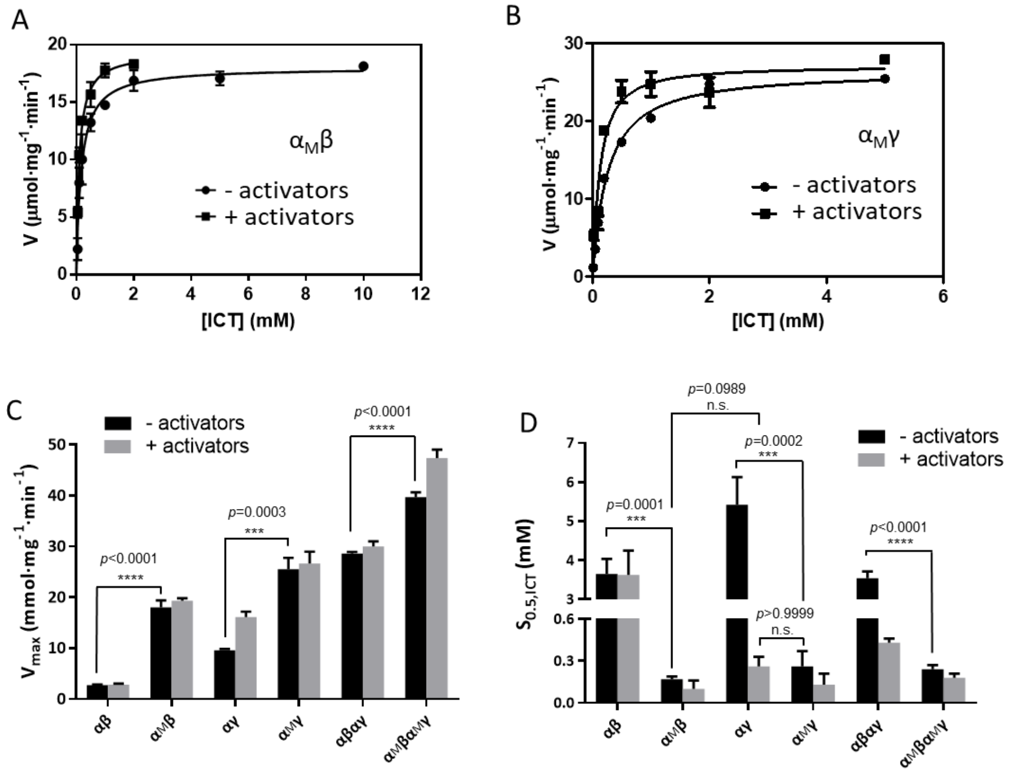

Figure S2. Catalytic properties of the WT and mutant HsIDH3. (A-B) Enzymatic activities of $\alpha_M\beta$ (A) and $\alpha_M\gamma$ (B) in the absence and presence of activators (ADP and CIT). Experiments were performed in triplicates. Data are presented as the mean ± SD. (C-D) Comparison of V_{max} (C) and $S_{0.5,ICT}$ (D) of the WT and mutant HsIDH3 in the absence and presence of activators. Bar graphs are generated with data presented in Table 1. Data are presented as the mean ± SD. Statistical analysis was performed by unpaired Student's t test. $^{*}p < 0.05$; $^{**}p < 0.01$; $^{***}p < 0.001$; $^{****}p < 0.0001$; n.s., not significant.

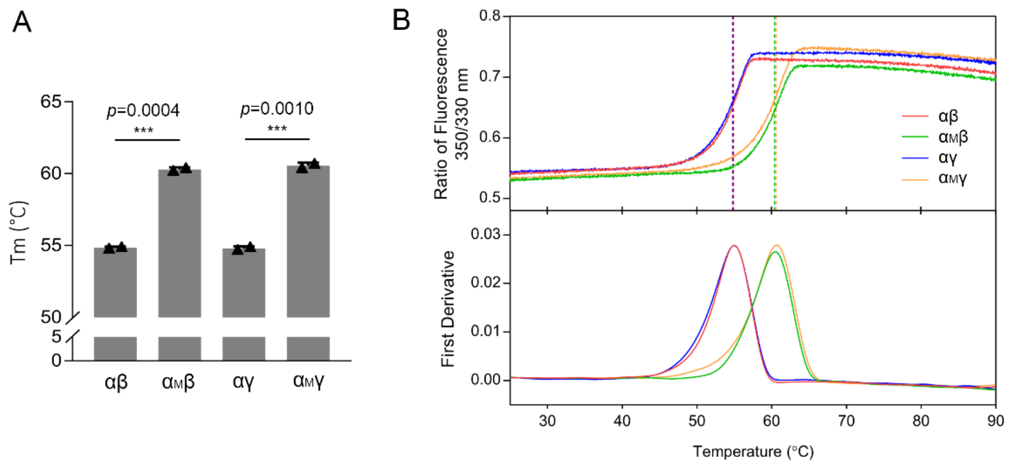

Figure S3. Thermal shift assay (TSA) of αβ, αγ, αMβ and αMγ. (A) Melting temperatures (Tm) of αβ, αγ, αMβ and αMγ. All proteins were analyzed at a concentration of 1 mg/mL. Experiments were done in duplicates. Data are presented as the mean ± SD. *** $p < 0.001$. (B) Representative melting curves of αβ, αγ, αMβ and αMγ (upper panel) and the curves of the first derivatives showing the Tm of each protein (lower panel).

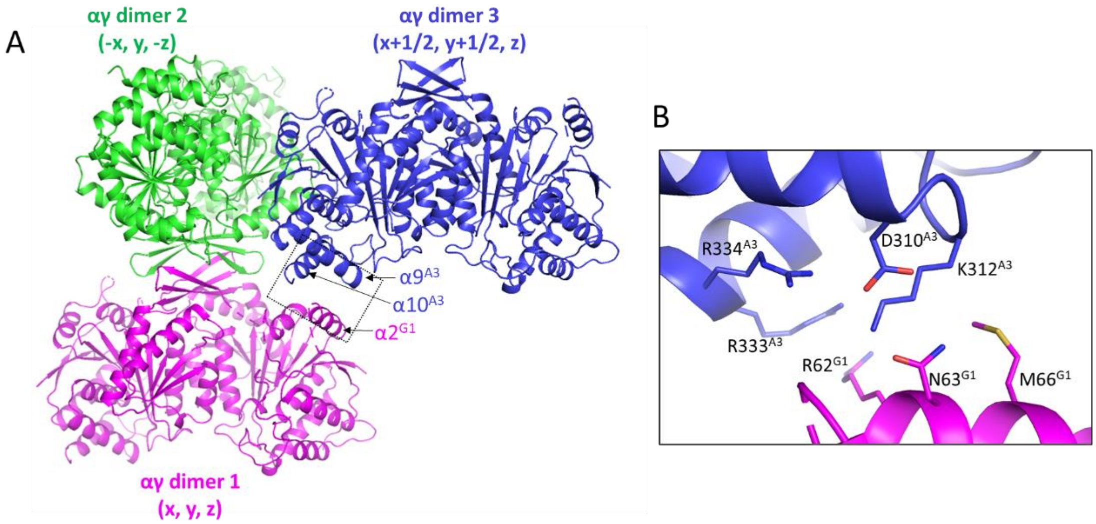

Figure S4. Crystal packing analysis of the αMγ structure reveals that the γ large domain is involved in close contacts with the α large domain of a symmetry-related heterodimer. (A) Heterodimer 1 and heterodimer 2 are related by crystallographic 2-fold axis; and heterodimer 1 and heterodimer 3 are related by a translational operation (1/2, 1/2, 0). The α2 helix of the γ subunit of heterodimer 1 makes close contacts with the α9 and α10 helices of the α subunit of heterodimer 3. (B) The zoom-in view of the contacting region between heterodimer 1 and heterodimer 3. Residues involved in close contacts (< 4 Å) between the two heterodimers are shown in stick model.

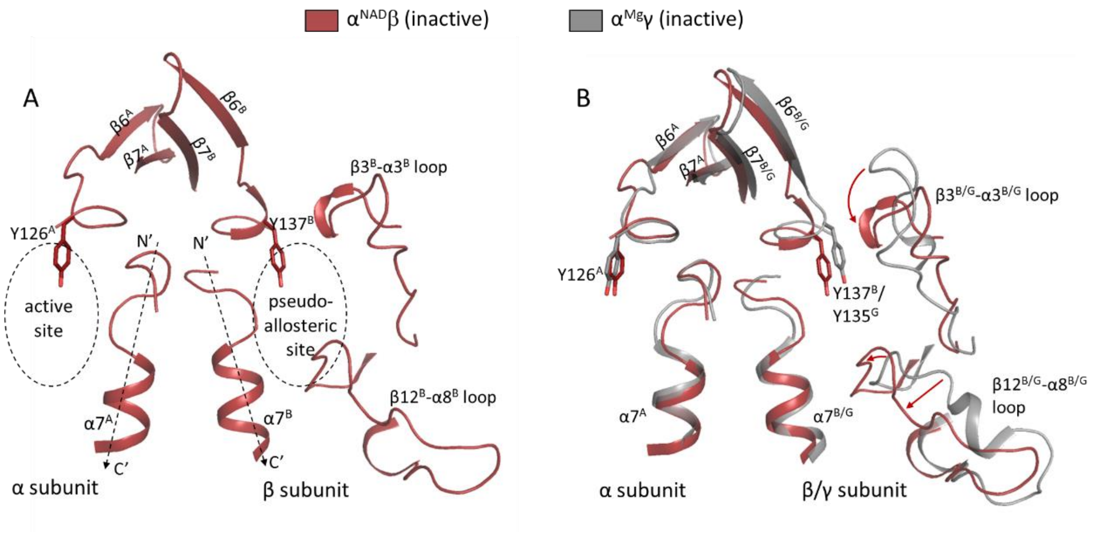

Figure S5. Structure of $\alpha^{NAD}\beta$ assumes inactive conformation. (A) The N-terminal regions of the $\alpha7$ helices and the $\beta5$-$\beta6$ loops of the $\alpha^{NAD}\beta$ structure adopt inactive conformations. The β pseudo-allosteric site is formed by the $\beta3^B$-$\alpha3^B$ and $\beta12^B$-$\alpha8^B$ loops. (B) Superposition of $\alpha^{NAD}\beta$ and $\alpha^{Mg}\gamma$ shows that the $\beta3^B$-$\alpha3^B$ and $\beta12^B$-$\alpha8^B$ loops adopt different conformations in the two structures.

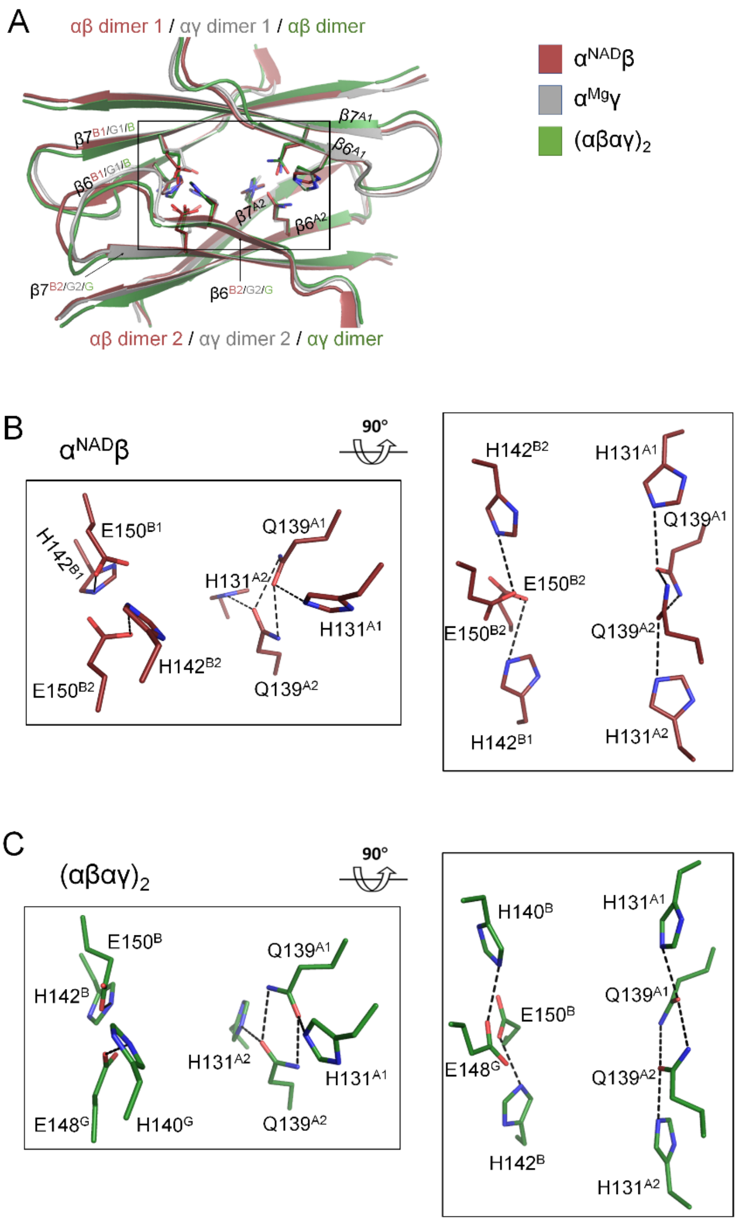

Figure S6. The heterodimer-heterodimer interfaces are similar in the structures of the inactive αβ and αγ heterodimers and $(\alpha\beta\alpha\gamma)_2$ holoenzyme. The crystal structures of the inactive $\alpha^{NAD}\beta$ (PDB 6KDY), $\alpha^{Mg}\gamma$ (PDB 5GRH) and $(\alpha\beta\alpha\gamma)_2$ (PDB 7CE3) are used as representatives. (A) Superposition of the clasp domains in the structures of the inactive $(\alpha\beta)_2$, $(\alpha\gamma)_2$ and $(\alpha\beta\alpha\gamma)_2$. The residues involved in the hydrogen bonding-network at the heterodimer-heterodimer interfaces are shown in stick model. (B) Zoom-in views of the hydrogen-bonding network at the αβ-αβ interface in the $\alpha^{NAD}\beta$ structure. (C) Zoom-in views of the hydrogen-bonding network at the αβ-αγ interface in the $(\alpha\beta\alpha\gamma)_2$ structure. Hydrogen bonds are indicated with dash lines.

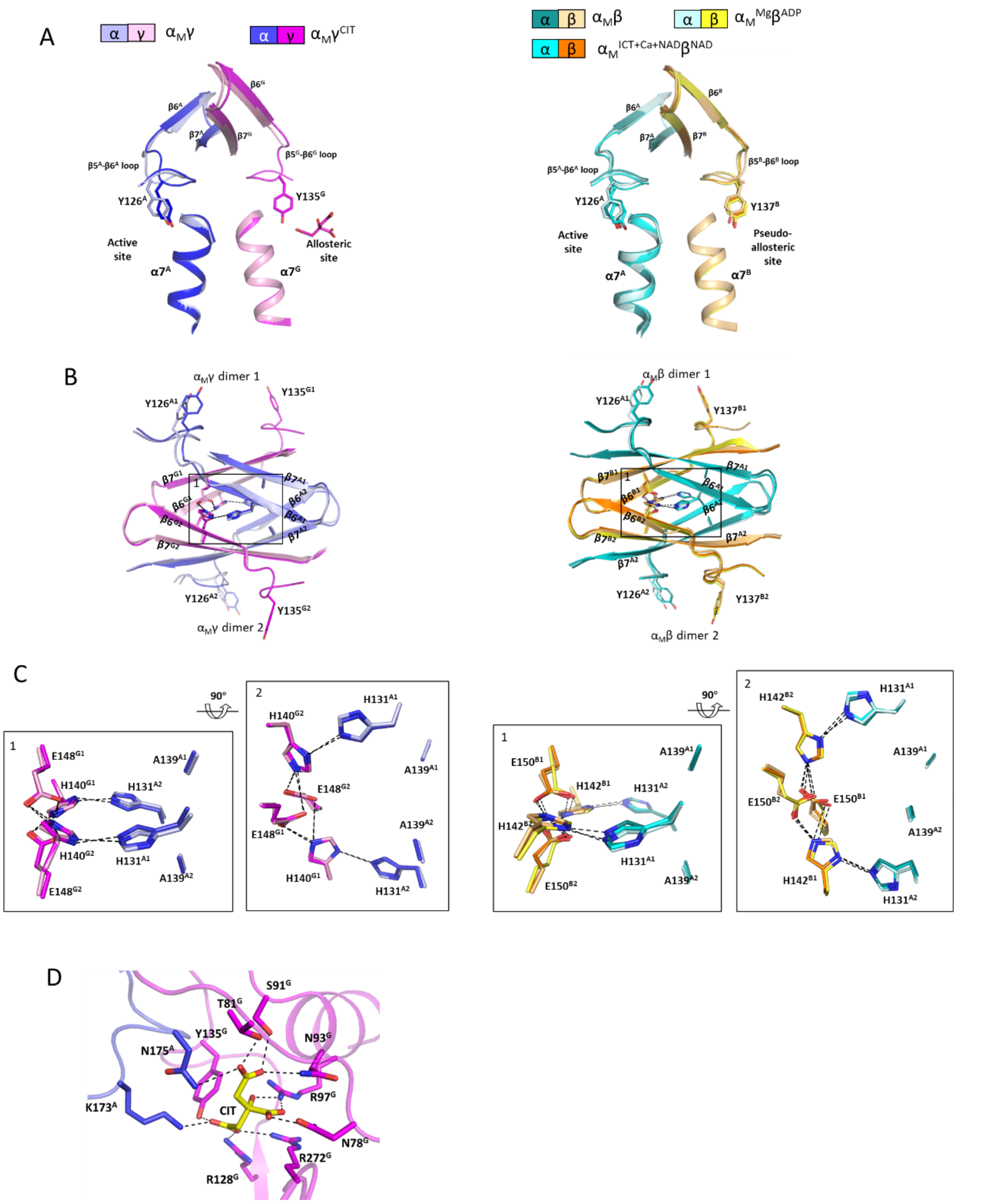

Figure S7. The overall structures of $\alpha_M\gamma$ and $\alpha_M\beta$ assume active conformations in both apo and ligand-bound forms. The structures of apo $\alpha_M\gamma$ and $\alpha_M\gamma^{CIT}$ are superposed (left panels), and the structures of apo $\alpha_M\beta$, $\alpha_M{}^{Mg}\beta^{ADP}$ and $\alpha_M{}^{ICT+Ca+NAD}\beta^{NAD}$ are superposed (right panels). (A) The α7 helices and β5-β6 loops in the $\alpha_M\gamma$ structures (left panel) and $\alpha_M\beta$ structures (right panel) assume active conformations. (B) The heterodimer-heterodimer interfaces in the $\alpha_M\gamma$ structures (left panel) and $\alpha_M\beta$ structures (right panel) are mediated by the clasp domains. (C) The hydrogen-bonding networks at the heterodimer-heterodimer interfaces in the $\alpha_M\gamma$ structures (left panel) and $\alpha_M\beta$ structures (right panel) assume active states. (D) The hydrogen-bonding interactions between CIT and the protein residues in the $\alpha_M\gamma^{CIT}$ structure. Hydrogen bonds are indicated by black dashed lines in all panels.

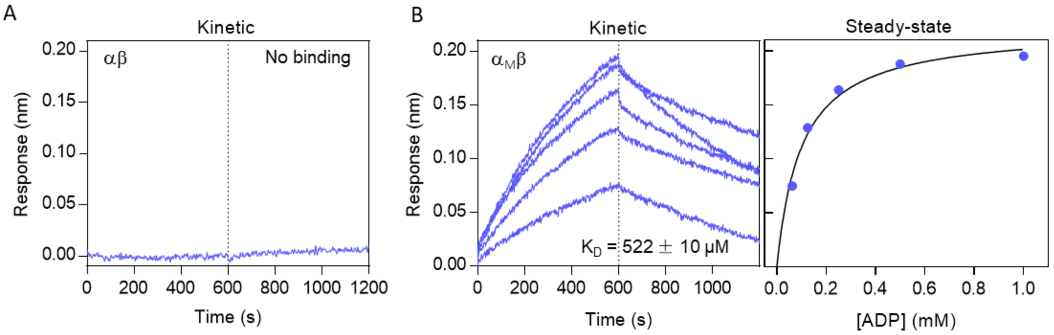

Figure S8. Bio-layer interferometry (BLI) analysis of ADP binding to αβ or $\alpha_M\beta$. (A) Association and dissociation curves of ADP (1 mM) show no binding to αβ. (B) Left: Association and dissociation curves of the ADP (62.5 μM - 1 mM) binding to $\alpha_M\beta$. Right: Steady state analysis of the ADP binding to $\alpha_M\beta$.

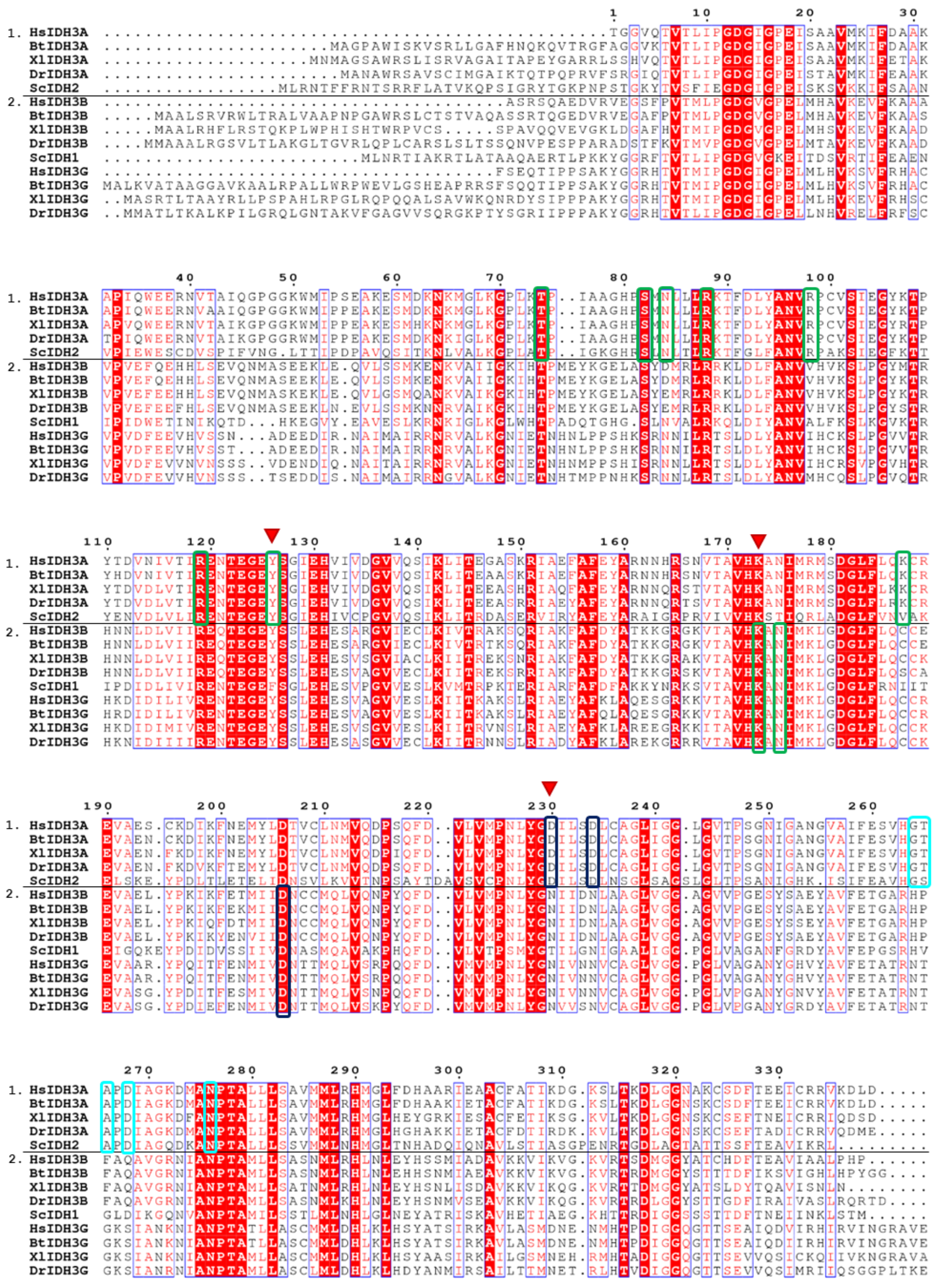

Figure S9. Comparison of HsIDH3 with the homologous NAD-IDH from *Bos taurus* (BtIDH3), *Xenopus laevis* (XlIDH3), *Danio rerio* (DrIDH3) and *Saccharomyces cerevisiae* (ScIDH). HsIDH3A, BtIDH3A, XlIDH3A, DrIDH3A and ScIDH2 are catalytic subunits, and the others are regulatory subunits. The residues involved in the hydrogen-bonding interactions with NAD (cyan boxes), ICT (green boxes) and metal ion (black boxes), and the residue triad responsible for the catalytic reaction (red triangle) are indicated.

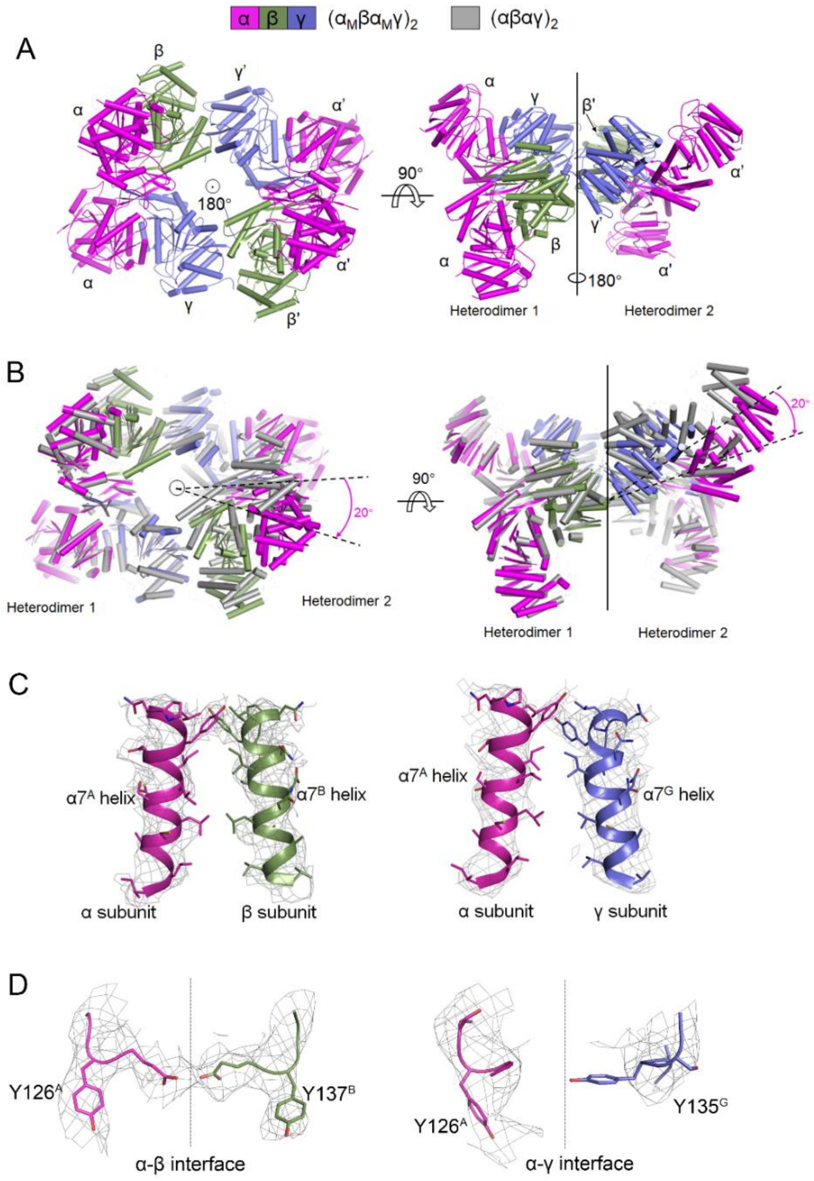

Figure S10. Structure of the unliganded ($\alpha_M\beta\alpha_M\gamma$)$_2$ holoenzyme assumes active conformation. (A) Overall structure of the ($\alpha_M\beta\alpha_M\gamma$)$_2$ holoenzyme. Two $\alpha_M\beta\alpha_M\gamma$ heterotetramers 1 and 2 are arranged in nearly perpendicular to each other with a distorted tetrahedron architecture to form the ($\alpha_M\beta\alpha_M\gamma$)$_2$ holoenzyme. (B) Comparison of the overall structures of the unliganded mutant ($\alpha_M\beta\alpha_M\gamma$)$_2$ holoenzyme (colored as in panel A) and the WT ($\alpha\beta\alpha\gamma$)$_2$ holoenzyme (colored in grey). When heterotetramer 1 of the two structures is superposed, heterotetramer 2 of the mutant holoenzyme shows approximately 20° rotation relative to that of the WT holoenzyme. (C) Composite omit map for the N-terminal regions of the α7 helices in the $\alpha_M\beta$ and $\alpha_M\gamma$ heterodimers. (D) Composite omit map for the sidechains of Tyr126^A and Tyr137^B in the $\alpha_M\beta$ heterodimer and the sidechains of Tyr126^A and Tyr136^G in the $\alpha_M\gamma$ heterodimer. Composite omit map is shown at a contour level of 1.0σ.
